# Epilepsy-Induced Reduction in HCN Channel Expression Contributes to an Increased Excitability in Dorsal, But Not Ventral, Hippocampal CA1 Neurons

**DOI:** 10.1523/ENEURO.0036-19.2019

**Published:** 2019-04-02

**Authors:** Elizabeth C. Arnold, Calli McMurray, Richard Gray, Daniel Johnston

**Affiliations:** 1 Institute for Neuroscience, University of Texas at Austin, Austin, TX 78712; 2 Center for Learning and Memory, University of Texas at Austin, Austin, TX 78712

**Keywords:** CA1 pyramidal neuron, dendrites, intrinsic properties, septotemporal axis, temporal lobe epilepsy, whole-cell electrophysiology

## Abstract

CA1 neurons in epileptic animals are vulnerable to selective changes in ion channel expression, called acquired channelopathies, which can increase the excitability of a neuron. Under normal conditions there is a gradient of ion channel expression and intrinsic excitability along the longitudinal, dorsoventral axis of hippocampal area CA1 of the rodent. Many of these channels, including M-channels, GIRK channels and HCN channels, all have dorsoventral expression gradients that might be altered in rodent models of epilepsy. Here, we show that the excitability of dorsal, but not ventral CA1 neurons, had an increased firing rate, reduced interspike interval (ISI) and increased input resistance in a status epilepticus (SE) model of temporal lobe epilepsy (TLE). As a result, the excitability of CA1 neurons became uniform across the dorsoventral axis of the rat hippocampus post-SE. Using current clamp recordings with pharmacology and immunohistochemistry, we demonstrate that the expression of HCN channels was downregulated in the dorsal CA1 region post-SE, while the expression of M and GIRK channels were unchanged. We did not find this acquired channelopathy in ventral CA1 neurons post-SE. Our results suggest that the excitability of dorsal CA1 neurons post-SE increase to resemble the intrinsic properties of ventral CA1 neurons, which likely makes the hippocampal circuit more permissible to seizures, and contributes to the cognitive impairments associated with chronic epilepsy.

## Significance Statement

Temporal lobe epilepsy (TLE) is characterized by spontaneous seizures. Evidence from patients and animal models suggest seizures are more likely to start in the ventral hippocampus. It is thought that disruptions in ion channel expression contributes to the generation of spontaneous seizures. In this study, we compared the intrinsic properties of neurons at either end of hippocampal area CA1 in chronic epilepsy and found that through a reduction in HCN channel expression the intrinsic properties of neurons in dorsal CA1 change to resemble the properties of ventral CA1 neurons. This work provides an anatomic context for the pathophysiological changes associated with epilepsy, and has implications for finding better treatments for epilepsy.

## Introduction

Temporal lobe epilepsy (TLE), affecting the hippocampus and surrounding cortices of the temporal lobe, is one of the most difficult forms of epilepsy to manage (Engel, 2001). Up to one third of patients have treatment-resistant, intractable TLE, which makes the affected networks susceptible to future seizures and cognitive impairment (Gowers, 1882; Helmstaedter and Kockelmann, 2006; French, 2007; Coan and Cendes, 2013).

In the clinical population and animal models, the hippocampus is not uniformly affected. In intractable TLE the anterior hippocampus is commonly targeted for surgical resection, and more vulnerable to cell loss compared to the posterior hippocampus (Wiebe et al., 2001; Schramm, 2008; Thom et al., 2012). Likewise in rodent models of epilepsy, neuronal loss, synaptic remodeling, and seizure initiation are more pronounced in the ventral (analogous to the human anterior hippocampus) compared to the dorsal hippocampus (Cavazos et al., 2004; Ekstrand et al., 2011; Toyoda et al., 2013).

Under normal conditions, the dorsal and ventral hippocampus have differences in connectivity, neuromodulatory tone, genetic expression markers and place field properties (Amaral and Witter, 1989; Fanselow and Dong, 2010; Strange et al., 2014). In addition, in area CA1 the physiological and morphologic properties of pyramidal neurons systematically differ along the dorsoventral axis of the hippocampus (Dougherty et al., 2012; Marcelin et al., 2012a; Hönigsperger et al., 2015; Malik et al., 2016; Milior et al., 2016). The differential distribution of ion channels like K_v_7/M, GIRK, and HCN in conjunction with differences in morphology across the dorsoventral axis contribute to the increased intrinsic excitability of ventral CA1 neurons, compared to dorsal neurons (Marcelin et al., 2012b; Dougherty et al., 2013; Hönigsperger et al., 2015; Kim and Johnston, 2015). These differences could contribute to the increased sensitivity of the ventral hippocampus to epileptogenic stimuli (Elul, 1964; Racine et al., 1977; Gilbert et al., 1985; Bragdon et al., 1986).

While the mechanisms of TLE are still not well understood, there is a clear association between the presence of seizures and the dysregulation of ion channel expression (Yus-Nájera et al., 2003; Heinzen et al., 2007; Aronica et al., 2009). In animal models of chronic epilepsy, CA1 neurons have been reported to have an increase in persistent sodium current and transient calcium current, but a reduction in the fast-inactivating A current, and hyperpolarization activated h current (Ketelaars et al., 2001; Su et al., 2002; Bernard et al., 2004; Jung et al., 2007; Shin et al., 2008; Jung et al., 2011).

It is unknown whether epilepsy-induced changes in neuronal excitability and the presence of acquired channelopathies are uniformly expressed across the dorsoventral axis of CA1. To address this question, we first examined the excitability phenotype of dorsal and ventral CA1 neurons in a post-status epilepticus (SE) model of TLE using whole-cell current clamp recordings. We found that post-SE the intrinsic membrane excitability of dorsal but not ventral CA1 neurons was increased. This resulted in the normally disparate intrinsic membrane properties along the dorsoventral axis of area CA1 to become uniform post-SE. We then tested the expression of three ion channel types using pharmacology and immunohistochemistry. We found that this increase in excitability co-occurred with a selective reduction in HCN but not M or GIRK ion channel expression. These data further strengthen the link between HCN channels and epilepsy, but show that this acquired channelopathy is not found in ventral CA1 neurons, an area typically associated with seizure initiation and hippocampal sclerosis. We hypothesize that through the reduction of HCN channels the excitability of dorsal CA1 neurons is increased, which makes dorsal CA1 neurons become more like ventral CA1 neurons, and thus are more prone to engage in epileptiform and seizure activity.

## Materials and Methods

### Animals

Male Sprague Dawley CD rats (Charles River) weighing 150–175 g were acclimated to the facility for one to two weeks after arrival. While in the facility, they were maintained on a 12/12 h light/dark cycle, and had access to food and water *ad libitum*. All procedures were done in accordance with the rules and regulations of the University of Texas at Austin Institutional Animal Care and Use Committee.

### Video-EEG recording

#### Surgery

Animals were implanted with electrodes and preamplifier on the top of the skull for *in vivo* EEG monitoring. Before the surgery, electrode wires were tinned with flux and all tools were sterilized with anprolene gas. On the day of the surgery rats were anesthetized with isofluorane (4% in medical grade oxygen) for the first 10 min and maintained on 1.5–2% isofluorane. Toe pinch reflexes and breathing were monitored throughout the procedure. The head was shaved and positioned into a stereotaxic frame. Using aseptic surgical techniques, a large incision was made along the midline. For the subdural surgeries, the surface of the skull was scored with a razor blade and nine holes were drilled for five subdural electrodes and four anchors. An EEG electrode was placed over each hippocampus (4 mm posterior to bregma, 4 mm lateral). The third EEG was placed over the frontal cortex 1 mm anterior to bregma, 1.5 mm lateral). The reference and ground electrodes were placed above the cerebellum (11 mm posterior to bregma, 2 mm lateral). For depth electrode recordings, 0.5 MΩ Parylene-C Insulated Tungsten Microelectrodes (A-M Systems) were placed unilaterally in a bundle targeting the dorsal, intermediate, and ventral hippocampus. For all surgeries, leads were then connected to a three EEG Headmount, which were all custom ordered so that each electrode was compared to a single reference electrode (Pinnacle Technologies). Dental cement was used to secure the headmount to the skull. Animals were given Rimadyl and Baytril to aid recovery.

#### Recording

After recovery baseline recordings were made, typically for ∼5 d after the surgery. Tethered recordings were made from individually housed rats. Rats had access to food, water and bedding during the recordings. EEG signals were acquired, amplified and digitized using the three EEG Sirenia system (Pinnacle Technologies). Data were acquired at 800 Hz and filtered at 400 Hz. Time synced video footage was also captured for each animal. v-EEG recording from one post-SE rat was continuous, while the others were intermittent (3 d/week; 8 h/d for two months). In all cases, seizures were detected *post hoc* using a 3-s sliding window with a 15-ms step size. When the power was calculated to be above 100 μV^2^, voltage traces were flagged for inspection. Only seizures with both a behavioral and electrographic component were counted.

### SE induction

Rats received a single intraperitoneal injection of 15-mg/kg kainic acid (Abcam) or a water vehicle. Within an hour after kainite behavioral seizures were apparent and rats began progressing through the Racine scale (0, no sign of seizure; 1, behavioral arrest; 2, head nodding; 3, forelimb clonus; 4, rearing; 5, rearing and falling; Racine, 1972). After the first class 5 seizure, rats remained in SE for 1 h. Animals that did not have a class 5 seizure were not included in the study. Seizures were terminated with a subcutaneous injection of 30-mg/kg pentobarbital (Sigma-Aldrich) prepared in ethanol, propylene glycol and water and sterile filtered. Following induction, rats were given a saline injection, wet food, and single housed for the remainder of the experiment.

### Slice preparation

Rats were anesthetized with an intraperitoneal injection of 90-mg/kg ketamine and 10-mg/kg xylazine and transcardially perfused with cold (∼4°C) oxygenated cutting saline containing: 210 mM sucrose, 7 mM dextrose, 2.5 mM KCl, 1.25 mM NaH_2_PO_4_, 25 mM NaHCO_3_, 7 mM MgCl_2_, 0.5 mM CaCl_2_, 1.3 mM ascorbate, and 3 mM pyruvate. The brain was then removed and the midline was cut. For dorsal slices, the hemisphere was blocked by making two cuts at 45° from the coronal plane at the anterior (above the striatum) and posterior (occipital cortex) ends of the forebrain. The tissue section was mounted on the posterior cut. The ventral hippocampus was prepared by making an oblique cut (∼15° from the horizontal plane) to the dorsal surface brain, and mounted on that cut surface. 350-µm slices were then made with a vibrating blade microtome (VT1000A, Leica Microsystems Inc.). Slices were transferred to a bubbled (95%O_2_, 5%CO_2_), heated (34°C) recovery chamber for 30 min, which contained: 125 mM NaCl, 2.5 mM KCl, 1.25 mM NaH_2_PO_4_, 25 mM NaHCO_3_, 2 mM MgCl_2_, 2 mM CaCl_2_, 10 mM dextrose, 1.3 mM ascorbate, and 3 mM pyruvate. Afterward the slices were kept at room temperature.

### Whole-cell patch clamp recordings

#### Recording configuration

For all recordings, slices were submerged in a heated, 32–34°C chamber, and perfused with bubbled artificial cerebral spinal fluid, which was perfused at 1–2 ml/min with artificial cerebral spinal fluid containing: 125 mM NaCl, 3 mM KCl, 1.25 mM NaH_2_PO_4_, 25 mM NaHCO_3_, 1 mM MgCl_2_, 2 mM CaCl_2_, 10 mM dextrose, and 3 mM pyruvate at pH ∼7.4. Slices were visualized on an Axioskop 2 (Carl Zeiss Microscopy) with differential interference contrast optics and an infrared video camera (DAGE-MTI). Healthy pyramidal neurons in the middle of the proximal-distal axis of CA1 were targeted. Data were acquired with a Dagan BVC-700A amplifier with a 0.1 N headstage (Dagan Corp.), and digitized with an ITC-18 (HEKA Instruments Inc.). Data in this study were acquired at 10–20 kHz and filtered at 3–10 kHz. The pipette capacitance was compensated and the bridge was balanced throughout all recordings. Series resistance was monitored through recordings, and ranged from 8–30 MΩ for somatic recordings and 13–35 MΩ for dendritic recordings. The liquid junction potential, estimated to be ∼12 mV, was not corrected.

#### Microelectrodes

Borosilicate capillary glass 1.65-mm external diameter (World Precision Instruments) was pulled with a Flaming/Brown micropipette puller (model P-97, Sutter Instruments). Electrodes used for somatic recordings were pulled to have a resistance of 4–6 MΩ. For dendritic recordings electrodes had a resistance of 6–9 MΩ and were wrapped with Parafilm to reduce the capacitance of the electrode. Electrodes were filled with a solution containing: 120 mM potassium gluconate, 8 mM NaCl, 16 mM KCl, and 11 mM HEPES, 4 mM Mg-ATP, 0.3 mM Na-GTP, 7 mM 2K-phosphocreatine, and 0.2% neurobiotin; pH 7.37. For dendritic recordings, 16 µM Alexa Fluor 594 (Thermo Fisher Scientific) was included to determine recording location.

#### Drugs

For all experiments, external AFSF saline included 20 µM 6,7 dinitroquinoxaline-2,3-dione (DNQX), 25 µM D-22-amino-5 phosphonovaleric acid (D-APV), which were obtained from Alomone Labs, and 2 µM SR-95531 (gabazine; Abcam). In some experiments, 10 µM ZD7288, 10 µM XE991, 2 µM CGP-55845, or 0.5 µM TTX was included in the ACSF (Abcam). In addition, for some experiments, 2 mM NiCl_2_ or 50 µM BaCl_2_ (Sigma-Aldrich) was included in the ACSF. ZD7288 was only introduced through the bath transiently, for 3–4 min. This prevented a nonspecific depolarization that occurs with continuous bath application, yet provided a stable block for ∼30 min (Kim and Johnston, 2015).

#### Acquisition and analysis of subthreshold measurements

Data were acquired and analyzed with a custom written software in Igor Pro (Wavemetrics). To measure the input resistance and rebound slope, a family of 800-ms-long current injections from –150–50 pA was delivered to the cell. Voltage traces were excluded from the analysis if action potentials were generated. To calculate the input resistance, the change in voltage was plotted against the current amplitude (–70–10 pA), and the slope of a linear fit was reported. The amplitude of the rebound depolarization was plotted against the membrane potential at the end of the step, and the slope of a linear fit was referred to as the rebound slope. To calculate the peak resonance frequency, a ±50-pA sinusoidal current that increased in frequency from 0 to 15 Hz over 15 s was injected to the cell. The current and the voltage response were then transformed from a times series into the frequency domain with a fast Fourier transform. A ratio of the real portion of the transformed voltage and current was used to calculate the impedance amplitude response. This relationship was fit with a polynomial function, and the frequency at which the impedance was at its maximum was defined as the peak resonance frequency.

#### Analysis of suprathreshold measurements

To measure the firing intensity a family of 800-ms-long current injections from 100 to 500 pA were delivered to the cell. The action potentials generated were measured at –20 mV. From traces that had 8–11 action potential, features of action potential shape were measured. The threshold was defined as the membrane potential at which the first derivative exceeded 20 mV/ms. The maximum of the first derivative was measured. The membrane potential at the peak of each action potential was subtracted from rest to calculate the amplitude. The fast afterhyperpolarization (fAHP) was detected by finding location within 1.5 ms of each spike where the derivative crossed zero for the second time. The membrane potential at this time was then subtracted from the threshold membrane potential to calculate the fAHP amplitude. The spike frequency accommodation (SFA) was reported as a ratio of the sixth spike to the first.

### *Post hoc* longitudinal position model

#### Histologic processing

During the physiological recordings, cells were filled with neurobiotin and were then fixed in 3% glutaraldehyde in 0.1 M phosphate buffer and stored at 4°C for 48 h for three months. These slices were then processed to amplify the biotin signal with avidin using an avadin HRP system, and a diaminobenzene precipitate was made for visualization (Vector Laboratories). Slices were mounted in glycerol. This was done for all cells to confirm that the slicing process did not damage the recorded neurons.

#### Dorsoventral statistical model

Images of processed slices were imported into FIJI (Schindelin et al., 2012). Two measurements were made in the transverse plane of the hippocampal subregions CA1, CA3, and DG. Within CA1, the transverse length of the pyramidal cell layer from CA2 to the subiculum was measured. In addition, the radial length of the distal dendritic layer, stratum lacunosum moleculare (SLM), was measured at the middle proximal-distal axis of CA1. A ratio of the transverse to the radial SLM lengths were calculated for CA1; this value differed most along the dorsoventral axis. In CA3 a ratio of the transverse length (including CA2) to the radial length, from the alveus to the hippocampal fissure, was calculated. In the dentate gyrus a ratio of transverse length including both blades, and the length from the tip of the infrapyramidal to the suprapyramidal blade was calculated. These ratios were then put into the linear regression model for rats: relative longitudinal position = –7.23 + 0.43(CA1 ratio) + 0.50(CA3 ratio) + 0.34(DG ratio). The rat hippocampus is estimated to be ∼10 mm long, and ∼8 mm of the entire structure has well-defined hippocampal subregions. Malik and colleagues segregated the longitudinal axis of the hippocampus into four 1.5-mm bins defined as dorsal, dorsal intermediate, ventral intermediate and ventral (Malik et al., 2016). The longitudinal location of a slice was predicted with an accuracy of ±0.59 mm with 90% confidence.

### Neuronal reconstructions

Filled cells that had a high signal-to-noise ratio were used for cellular reconstructions. Neuronal tracings were done under 40× magnification on a light microscope with the Neurolucida software (MBF Biosciences). Cell bodies and dendritic arbor were reconstructed, but spines and axons were not always visible, and therefore, were not included. Branching patterns were quantified using the Sholl analysis, where circles of increasing radii (20.6-μm increments) were overlaid on the neuron (Sholl, 1953). Dendritic length and surface area were computed using the Neurolucida software.

### Immunohistochemistry

#### Tissue preparation

Rats were anesthetized with an intraperitoneal injection of ketamine/xylazine and once anesthetized intracardially perfused with cold cutting saline (same as for acute slice preparation) and then 4% paraformaldehyde. Tissue was blocked for dorsal and ventral sections and then left in 4% PFA in 0.1 M phosphate buffer for 1 d. Tissue is then left at room temperature in a solution containing 30% sucrose and 2% PFA in 50 mM phosphate buffer for 3 d. Saturated tissue was sectioned to 50 µm on a freezing sliding microtome or cryostat (Leica Microsystems) and stored in cryoprotectant (50 mM phosphate buffer with 1.7 M glucose and 9.6 M glycol, pH 7.4).

#### Immunostaining

Free floating sections were rinsed with PBS, permeabilized with 0.5% Triton X-100 (Sigma-Aldrich), and incubated in a blocking buffer containing 0.25% Triton X-100 and 10% normal goat serum (Jackson ImmunoResearch) in PBS. Primary antibodies for channel subunit was multiplexed with mouse MAP2 (1:1000, M9942, Sigma-Aldrich) and incubated with the slices at 4°C overnight. The primary antibodies included HCN1 (1:500, RRID:AB_2115181, UC Davis/NIH NeuroMab Facility, Davis, CA), GIRK2 (1:200, APC-006, Alomone Labs), and K_V_7.2 (1:50, RRID:AB_2131704, UC Davis/NIH NeuroMab Facility). Primary antibody concentrations were compared to no antibody control, and concentrations were selected from a pilot experiments with three serial dilutions. Slices were then incubated with fluorophore-conjugated secondary antibodies complementing the hosts of the primary antibodies (1:500, Jackson ImmunoResearch). Sections were mounted in Fluoromount-G (Southern Biotech).

#### Data collection and analysis

Sections were visualized with a Zeiss Axio Imager Z2 microscope running AxioVision software (Carl Zeiss Microscopy). Images were acquired as multi-channel mosaics in 16-bit grayscale format. Exposure times were selected to prevent saturated pixels, and be under 450 ms for all experiments. Within an experiment the exposure time was uniform across all sections. Each image in an experiment was calibrated to the same scale, where 0 is black and a lighter signal is indicative of more protein. In FIJI, the average gray value was measured from images using either a rectangular box or the plot profile function (Schindelin et al., 2012). With the plot profile, the somatodendritic length was normalized and binned into 20 segments.

### Statistics

All data are represented as the mean ± SEM. Prism 7 (GraphPad) was used for all statistical analysis. In all cases the representative examples were the closest replicate to the group mean. Subthreshold properties were compared using *t* test, or ANOVA. Suprathreshold properties were compared with multiple *t* test corrected for multiple comparisons using the Holm–Sidak method. Reconstructions were compared using a *t* test or repeated measures two-way ANOVA. Immunohistochemical results were compared using a *t* test or multiple *t* tests with the Holm–Sidak correction.

## Results

### Animals have seizures within one-month post-SE

**Figure 1.**
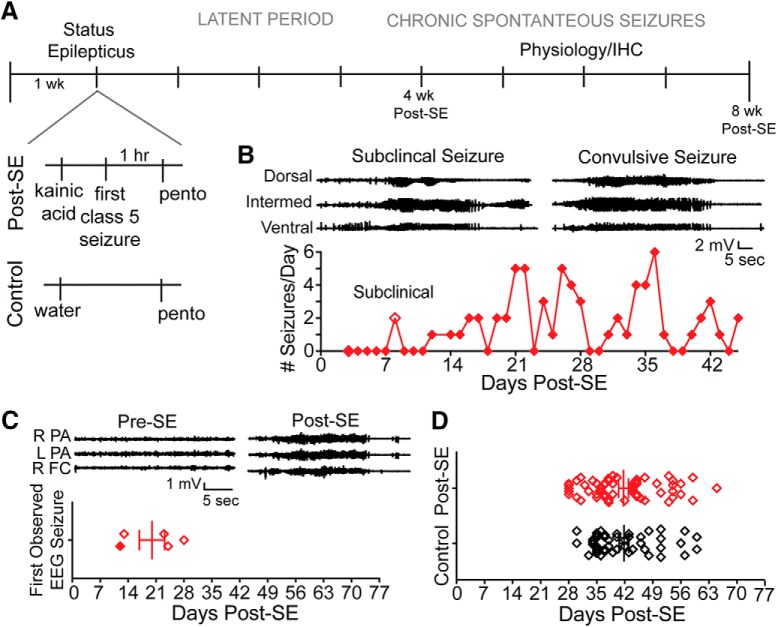
Spontaneous seizures occur within the first month after SE. ***A***, Description of SE protocol. Expansion below to the left illustrates the only difference between the treatment groups was an injection of kainic acid (post-SE) or vehicle (control). All rats, including controls, received an injection of pentobarbital. After status, post-SE rats underwent a variable length latent period, and then chronic spontaneous seizures begin. Rats were euthanized one to two months after the day of SE induction and used for physiology or immunohistochemistry experiments. ***B***, Example EEG from video-EEG monitoring of post-SE rat implanted with depth electrodes targeting the dorsal, intermediate, and ventral hippocampus. The first seizures were subclinical and occurred on day 8. The first electrographic seizures that also had a behavioral correlate, which were confirmed by video monitoring, occurred on day 12. The electrographic signature of these seizures is shown at the top. Below, the seizure frequency is shown through day 45 post-SE. ***C***, Representative EEG from an animal implanted with subdural electrodes positioned above the left and right parietal association cortex (L PA and R PA, respectively) and right frontal cortex (R FC). Video-EEG monitoring showed that before kainic acid injections, seizures were never observed. Pre-SE traces were taken from a period of quiet wakefulness, and traces to the right (post-SE) show the first observed convulsive seizure 13 d post-SE. Four rats were equipped with subdural electrodes and monitored 3 d/week for 8 h/d for at least the first two months post-SE. One animal was monitored continuously (from ***B***, filled diamond). Seizures were observed in the first month post-SE in all rats. The day of the first observed convulsive seizure for animals is plotted. ***D***, Days after injection (post-SE or control) is plotted for animals used in these studies.

After one week of habituation, rats randomly received an intraperitoneal injection of 15-mg/kg kainic acid or vehicle (Fig. 1*A*). The induced seizures were progressive and stereotyped as described by the Racine scale (Racine, 1972). Animals spent 1 h in SE, and were then given 30-mg/kg subcutaneous pentobarbital. Control rats also received an injection of pentobarbital to account for any possible effects of the compound. After the induced seizures, rats recovered and underwent a seizure-free latent period, and then began experiencing chronic recurrent seizures (Fig. 1*B*). Intermittent recordings from rats equipped with subdural cortical screws showed that seizures were detected within the first month (mean 20 ± 3.18 d; Fig. 1*C*), whereas no seizures were recorded from control animals (*n* = 2; data not shown). Since all animals implanted with EEG headstages had seizures in the first month (*n* = 5), subsequent electrophysiological or immunohistochemical experiments were conducted in the second month post-SE. Rats were euthanized for electrophysiological and biochemical experiments on average 42 d after the induction of SE (control: 41.8 ± 8.1 d, post-SE: 41.7 ± 9.3 d; Fig. 1*D*).

### Dorsal and ventral slices are from clearly defined regions along the longitudinal hippocampal axis

**Figure 2.**
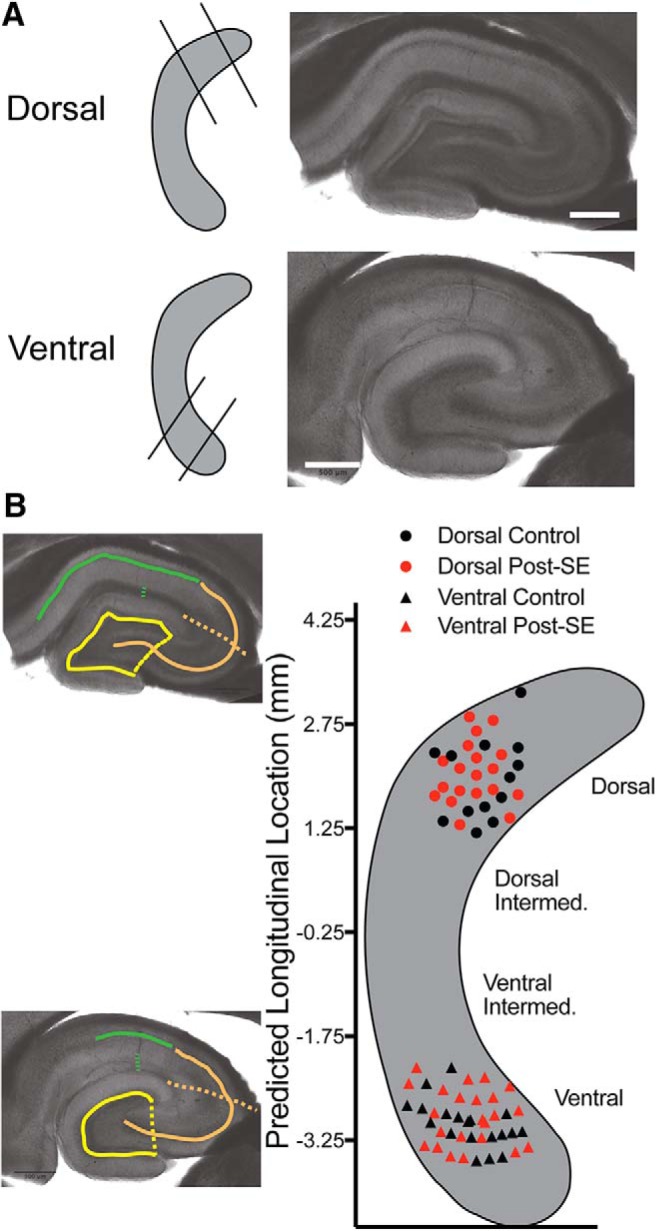
Slices were collected from identifiable and distinct regions of the hippocampus. ***A***, Preparation of all slices used in the following experiments targeted either the dorsal or the ventral hippocampus. To the left, a schematic of the blocking cuts used to collect slices from the dorsal (top) or ventral (bottom) hippocampus. To the right, representative sections show differences in hippocampal microarchitecture at either end of the dorsoventral axis. Note the distinctive difference in the shape of the dentate granule cell layer. ***B***, Dorsoventral location of slices were mapped on to the longitudinal axis of the hippocampus *post hoc*. Left, Representative images from above are overlaid with solid and dotted lines. These lines reflect measurements made from transverse hippocampal sections. A ratio of the length of the lines (solid/dotted) in hippocampal subregions CA1 (green), CA3 (orange), and DG (yellow) were put into the statistical model described in the text to estimate dorsoventral location. Right, Summary of predicted slice location along the dorsoventral axis. Symbols reflect location (e.g., circles are dorsal, and triangles are ventral hippocampus). Data from control rats are presented in black, and data from post-SE rats are presented in red. This color scheme is consistent throughout the manuscript.

We restricted our study to the poles of the longitudinal, dorsoventral axis of CA1. Representative dorsal and ventral sections are shown (Fig. 2*A*). We estimated the longitudinal location of 73 of the 88 slices used for the somatic recordings using a *post hoc* algorithm (Malik et al., 2016). We found slices intended to be collected from the dorsal and ventral hippocampus were, in fact, mapped back to the targeted locations (Fig. 2*B*).

More specifically, the predicted locations of the 8 mm of length of the hippocampus ranged from 4 mm, the dorsal-most location, to –4 mm, the most ventral. The predefined midpoint for the dorsal region was 2 mm, and the ventral region had a midpoint of –2.5 mm (Malik et al., 2016). We found that dorsal slices in this study had a midpoint of 2.01 ± 0.09 mm. The most dorsal recording had a predicted location of 3.21 mm and the most intermediate having a location of 1.19 mm. For ventral hippocampal slices, the predicted location was –2.95 ± 0.06 mm, with the most ventral recording having a predicted location of –3.54 mm and the most intermediate having a location of –2.19 mm. By employing the statistical model developed by Malik and colleagues we have shown that the following experiments were done in well-defined regions that are consistent with previous work (Fig. 2*B*). This is something that will be of increasing importance as our understanding of the hippocampus evolves.

### Dorsoventral differences in excitability are absent post-SE

**Figure 3.**
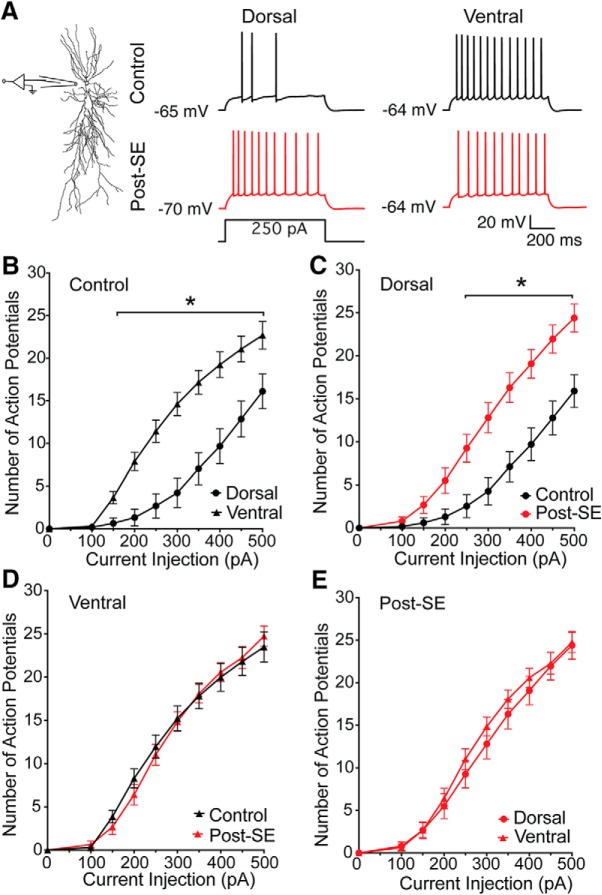
Dorsoventral difference in firing output is absent post-SE. ***A***, To the left the schematic shows the recordings location. To the right representative action potential trains evoked from the natural resting membrane potential with an 800-ms-long 250-pA current injection. ***B***, The firing intensity, which is the number of action potentials generated as a function of the amplitude of injected current, is plotted for current steps between 0 and 500 pA. The firing intensity obtained from whole-cell recordings of dorsal (circles) and ventral (triangles) CA1 pyramidal neurons from control animals are plotted. When we compared these group in *post hoc* comparisons we found that they were different for all current steps above 150 pA. ***C***, To compare the effect of epilepsy within regions, we plotted the firing intensity of dorsal CA1 neurons from control (black) and post-SE (red) rats. ***D***, Firing intensity of ventral CA1 neurons from control and post-SE rats were not statistically different at any current injection. ***E***, Firing intensity of dorsal and ventral CA1 neurons from post-SE rats. Data are expressed as the mean ± SEM. Statistical significance (*) is defined as *p* < 0.05.

Several labs have reported differences in the firing pattern of dorsal and ventral CA1 neurons (Dougherty et al., 2012; Marcelin et al., 2012a; Hönigsperger et al., 2015; Malik et al., 2016; Milior et al., 2016). In adult rats, CA1 pyramidal neurons in the ventral hippocampus fire more action potentials than CA1 neurons in the dorsal hippocampus in response to identical current injections. To test whether this dorsoventral difference in firing was present post-SE, whole-cell current clamp recordings were made at the resting membrane potential from CA1 pyramidal neurons in acute dorsal and ventral hippocampal slices from control and post-SE rats (Fig. 3*A*). In all experiments, blockers of fast synaptic transmission were used to detect only changes in excitability intrinsic to the cell being recorded. Treatment groups in Figure 3*B–E* are displayed separately for clarity, but were tested for statistical significance together (RM two-way ANOVA *F*_(24,576)_ = 6.461, *p* < 0.0001). Consistent with previous work, ventral CA1 neurons from control rats fired more action potentials than dorsal CA1 neurons (Tukey *post hoc*, *p* = 0.004 to <0.0001 for 200–500 pA; dorsal: 14 cells/10 rats, circles; ventral: 21 cells/16 rats, triangles; Fig. 3*B*). We then tested whether the firing of dorsal or ventral CA1 neurons changed post-SE. We found dorsal CA1 neurons had an increased firing output (Tukey *post hoc*, *p* = 0.001 to <0.0001 for 250–500 pA; Fig. 3*C*), but the firing pattern of ventral CA1 neurons was unchanged post-SE (Tukey *post hoc* comparisons, *p* = 0.91–0.99; Fig. 3*D*). This change in the firing of dorsal CA1 neurons post-SE resulted in an equivalent firing output of dorsal and ventral neurons post-SE (Tukey *post hoc* comparisons, *p* = 0.70 to >0.99; dorsal: 21 cells/16 rats; ventral: 20 cells/17 rats; Fig. 3*E*).

**Figure 4.**
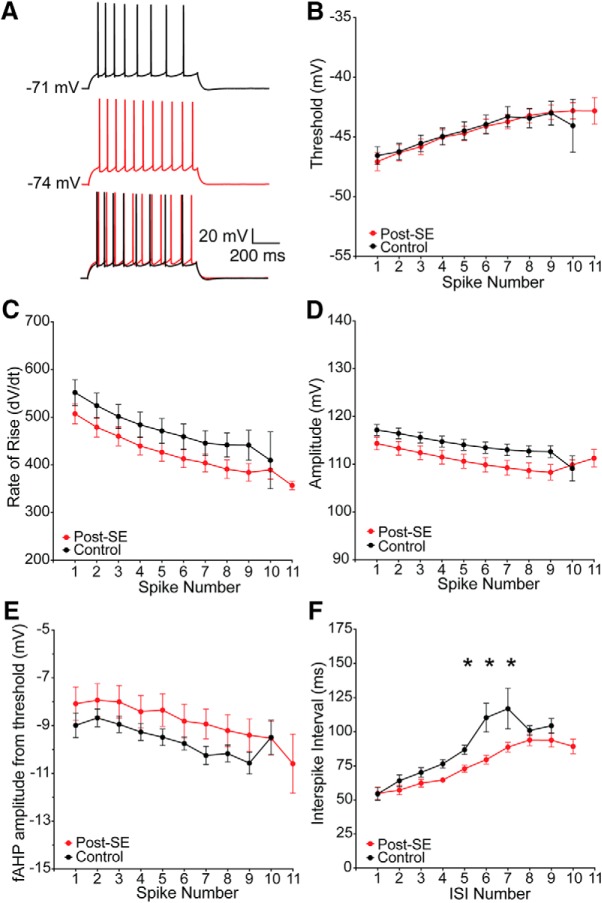
Reduced ISI contributes to increased firing in dorsal CA1 neurons post-SE. ***A***, Representative trains of 8–11 action potentials from dorsal CA1 neurons from control and post-SE groups. Trains are evoked from the resting membrane potential with a variable amplitude current injection. The traces are overlaid at the bottom, which allows for direct comparison of trace features. ***B***, Threshold, plotted as a function of spike number in the train, progressively increases, but was not different between the two groups. ***C***, Maximum rate of rise was also not different between the two groups. ***D***, Action potential amplitude was also not different between the two groups. ***E***, The amplitude of the fAHP immediately following repolarization was also measured. The amplitude was not different between the control and post-SE dorsal neurons. ***F***, ISI was measured between each action potential. Control dorsal neurons had a characteristic delay in the middle of the train, which was absent post-SE. *p < 0.05.

The generation of spike trains is dependent on the subthreshold and suprathreshold properties of a neuron. We first hypothesized that the increased firing in dorsal neurons post-SE resulted from a change in the repetitive spiking behavior. Trains that had 8–11 action potentials, in the middle of the response curve, were selected for further analysis. There was no difference the shape of the first action potential or the progression of action potentials between control and post-SE dorsal neurons (control: *n* = 14 cells/*N* = 11 rats; post-SE: *n* = 18 cells/*N* = 14 rats; Fig. 4*A*). The threshold (multiple *t* tests using Holm–Sidak correction, *p* < 0.99; Fig. 4*B*), rate of rise (multiple *t* tests using Holm–Sidak correction, *p* = 0.67–0.74; Fig. 4*C*), amplitude (multiple *t* tests using Holm–Sidak correction, *p* = 0.47–0.78; Fig. 4*D*), and fAHP (multiple *t* tests using Holm–Sidak correction, *p* = 0.66–0.98; Fig. 4*E*) were unchanged in dorsal neurons post-SE. The interspike interval (ISI), however, was reduced in the middle of the train in dorsal CA1 neurons post-SE (multiple *t* tests using Holm–Sidak correction, *p* = 0.02 ISI 4 and 5, *p* = 0.03 ISI 6; Fig. 4*A*,*F*).

**Figure 5.**
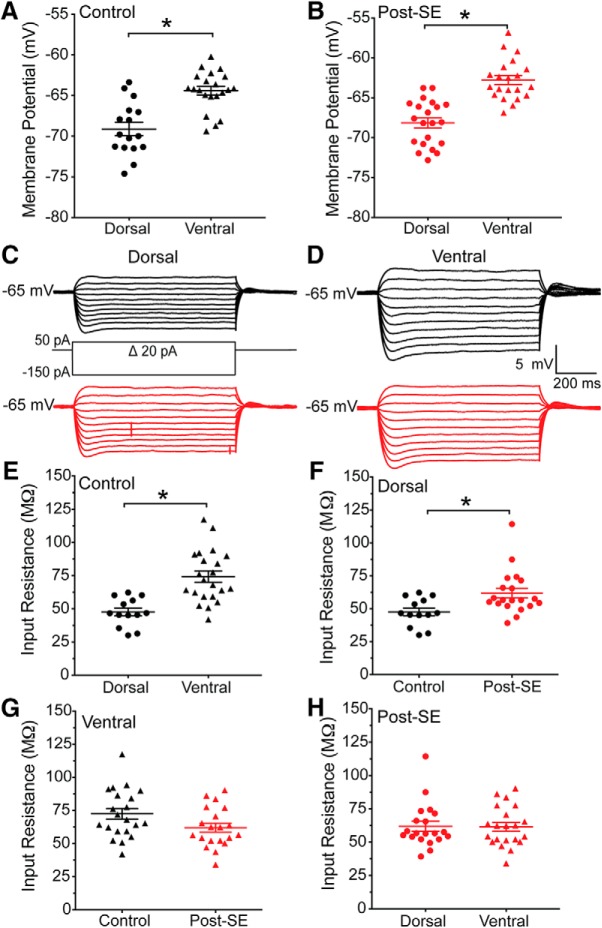
Input resistance is increased in dorsal CA1 neurons post-SE. ***A***, Dorsal and ventral cells had different resting membrane potentials in recordings from control rats. ***B***, Resting membrane potentials of dorsal and ventral CA1 neurons from post-SE rats are plotted. ***C***, Current injections delivered to neurons were 800-ms-long steps ranging from –150 to 50 pA. Representative voltage traces from dorsal CA1 neurons from control (black) and post-SE (red) groups are shown. Neurons were held at –65 mV. ***D***, Representative voltage traces recorded from ventral CA1 neurons in control and post-SE groups are shown. ***E***, Dorsal-ventral comparisons of input resistance from control rats are plotted. ***F***, The input resistance of dorsal CA1 neurons from control and post-SE groups are plotted. ***G***, Within ventral CA1, measurements of input resistance from control and post-SE neurons were plotted. ***H***, Dorsal-ventral comparisons of input resistance from post-SE rats are plotted. *p < 0.05.

Dorsal and ventral CA1 neurons have different subthreshold membrane properties in untreated rats (Dougherty et al., 2012; Hönigsperger et al., 2015; Malik et al., 2016). We measured the resting membrane potential of dorsal and ventral CA1 neurons from control and post-SE rats. In Figure 5, panels *A* and *B* are plotted separately to emphasize the role epilepsy played in setting resting membrane potential, but these data were tested for statistical significance collectively (one-way ANOVA *F*_(3,75)_ = 23.08, *p* < 0.0001). In CA1 neurons from control rats, dorsal neurons are on average 4.5 mV more hyperpolarized than ventral neurons, in agreement with previous reports (dorsal: –68.9 ± 0.9 mV, *n* = 16 cells/*N* = 10 rats; ventral: –64.4 ± 0.5 mV, *n* = 21 cells/*N* = 15 rats, Sidak *post hoc*, *p* < 0.0001; Fig. 5*A*). We found that in dorsal and ventral CA1 neurons post-SE this difference is preserved; dorsal neurons are on average 5.4 mV more negative than ventral neurons post-SE (dorsal: –68.2 ± 0.6, *n* = 21 cells/*N* = 14 rats; ventral: –62.8 ± 0.6 mV, *n* = 21 cells/*N* = 16 rats, Sidak *post hoc*, *p* < 0.0001; Fig. 5*B*). We next sought to measure input resistance, an indirect measure of ion channels open in the membrane at rest, and as such is dependent on the membrane potential. Similarly, data presented in Figure 5*E–H* are graphed separately to highlight specific relationships between the groups, but statistical comparisons were made collectively (ANOVA *F*_(3,70)_ = 8.56, *p* < 0.0001). In recordings from control dorsal and ventral CA1 neurons, we found, consistent with previous reports, at –65 mV the input resistance was larger in ventral CA1 neurons than dorsal CA1 neurons (dorsal: 46.1 ± 2.7 MΩ *n* = 13 cells/*N* = 10 rats; ventral: 73.5 ± 3.7 MΩ 21 cells/15 rats, Sidak *post hoc*, *p* < 0.0001; Fig. 5*C–E*). We then compared the effect of epilepsy on dorsal and ventral CA1 neurons. Dorsal CA1 neurons post-SE had a larger input resistance (Sidak *post hoc*, *p* = 0.02; Fig. 5*F*), but ventral CA1 neurons post-SE were not statistically different from control (Sidak *post hoc*, *p* = 0.08; Fig. 5*G*). To understand the relationship between dorsal and ventral neurons post-SE, we compared the input resistance of these neurons, and found that there was no difference between the input resistance of dorsal and ventral CA1 neurons post-SE (dorsal: 61.95 ± 3.7 MΩ *n* = 20 cells/*N* = 14 rats, ventral: 62.01 ± 3.7 MΩ 20 cells/16 rats; Sidak *post hoc*, *p* > 0.99; Fig. 5*H*).

**Figure 6.**
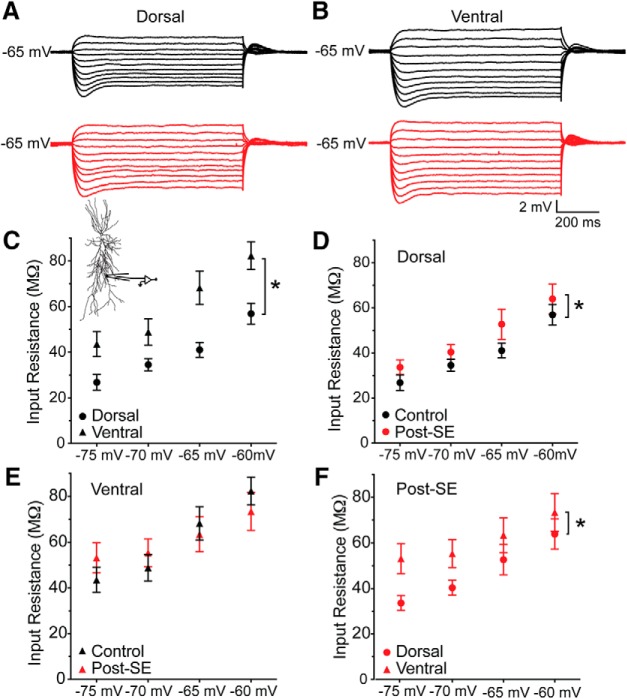
Dendritic input resistance is increased in dorsal CA1 neurons post-SE. ***A***, ***B***, Representative voltage traces from 800-ms-long current steps from –150 to 50 pA in dorsal (***A***) and ventral (***B***) CA1 neurons from control (black) and post-SE (red) groups. Dendrites were held at –65 mV. ***C***, Input resistance was calculated from families of traces collected at –75, –70, –65, and –60 mV for dorsal and ventral neurons from control animals. Inset shows recording location. ***D***, The input resistance of dorsal dendrites from control and epileptic animals were significantly different from each other. ***E***, Epilepsy as a factor did not have a significant effect in describing the variance of the input resistances of ventral dendrites. ***F***, The input resistance of dorsal and ventral neurons from post-SE animals are plotted. *p < 0.05.

Epilepsy-induced dysregulations in ion channel expression enriched in the dendrites have been reported (Bernard et al., 2004; Jung et al., 2007; Shin et al., 2008). In addition, there are differences between the dendritic input resistance of dorsal and ventral neurons (Dougherty et al., 2012). We wanted to test whether the intrinsic properties at the apical dendrite of dorsal and ventral CA1 neurons had changed post-SE. Whole-cell current clamp recordings were obtained from the apical dendrite of dorsal and ventral CA1 neurons. Since ventral neurons have a longer radial length than dorsal neurons, a slightly more distal location was targeted. The average recording location for dorsal neurons was 177 ± 11.7 µm from the soma, and for ventral neurons 207 ± 11.7 µm from the soma. After obtaining quality recordings we measured the voltage response to subthreshold current injections at four membrane potentials: –60, –65, –70, and –75 mV. Representative voltage traces from –65 mV are shown in Figure 6*A*,*B*. Under control conditions, the dendritic input resistance of ventral CA1 neurons is larger than dorsal CA1 neurons (two-way ANOVA *F*_(1,37)_ = 32.39, *p* < 0.0001; Fig. 6*C*). In addition, the input resistance of ventral CA1 neurons had a steeper voltage dependence than dorsal CA1 neurons. In dorsal CA1 neurons, the elevation of the dendritic input resistance post-SE is significantly different from controls (control: *n* = 7 dendrites/*N* = 6 rats, post-SE: *n* = 8 dendrites/*N* = 8 rats; two-way ANOVA *F*_(1,47)_ = 4.80, *p* = 0.03; Fig. 6*D*). In ventral CA1 neurons the dendritic input resistance is unchanged post-SE (control: *n* = 7 dendrites/*N* = 6 rats, post-SE: *n* = 8 dendrites/*N* = 7 rats, two-way ANOVA *F*_(1,44)_ = 0.01, *p* = 0.91; Fig. 6*E*). Post-SE the dendritic input resistance differed between dorsal and ventral neurons (two-way ANOVA *F*_(1,54)_ = 8.55, *p* = 0.005; Fig. 6*F*).

### Neuronal morphology is unchanged in post-SE model

**Figure 7.**
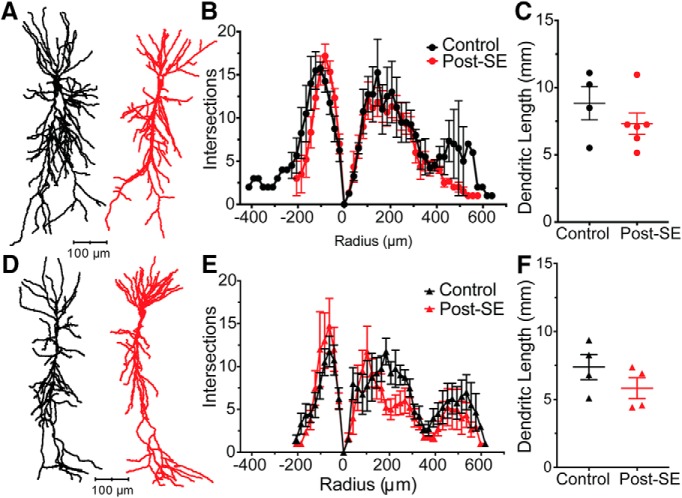
Cellular morphology unchanged in dorsal and ventral CA1 neurons post-SE. ***A***, Representative morphologic reconstructions of filled dorsal CA1 neurons from control and post-SE groups. ***B***, Branching pattern of dorsal neurons quantified with Sholl analysis where each concentric circle increased by 20.3 µm. ***C***, Total dendritic length in dorsal neurons from both control and post-SE groups are plotted. ***D***, Representative neuron tracings of ventral CA1 neurons from control and post-SE groups. ***E***, Sholl analysis quantified branching of the dendritic arbor in ventral CA1 neurons. ***F***, The dendritic length in ventral CA1 neurons in control and post-SE were not statically different.

In experimental epilepsy models, neuronal morphology has been reported to change (Pyapali and Turner, 1994; Drakew et al., 1996; Isokawa, 2000). The cellular response appears to be dependent on both the cell type and model used. As such, it is unknown whether the morphology of dorsal or ventral CA1 neurons change post-SE. This is of crucial importance to the interpretation of our experiments because the intrinsic properties of a neurons are determined by both the ion channel composition and distribution throughout a neuron, but also neuronal morphology. A subset of neuronal fills from fixed slices were traced under light microscopy using Neurolucida [control: *n* = 4 neurons/*N* = 4 rats; post-SE: *n* = 6 neurons/*N* = 6 rats (Fig. 7*A*); control: *n* = 4 neurons/*N* = 4 rats; post-SE: *n* = 4 neurons/*N* = 4 rats (Fig. 7*D*)]. Analysis of the branching pattern in dorsal (RM ANOVA, *F*_(26,208)_ = 1.33, *p* = 0.14; Fig. 7*B*) and ventral CA1 (RM ANOVA, *F*_(31,186)_ = 1.13, *p* = 0.30; Fig. 7*E*) neurons did not reveal an epilepsy-induced change in neuron shape. Further analysis of the total length of the dendritic arbor were not different in dorsal or ventral CA1 neurons [control: 8.84 ± 1.23 mm, post-SE: 7.32 ± 0.80 mm, unpaired *t* test, *p* = 0.31 (Fig. 7*C*); control: 7.38 ± 0.92 mm, post-SE: 5.82 ± 0.77 mm, unpaired *t* test, *p* = 0.24 (Fig. 7*F*)]. These cellular reconstructions suggested that a change in morphology cannot explain the changes in excitability post-SE.

In summary, under normal circumstances dorsal and ventral CA1 neurons have distinct intrinsic properties due to differences in the distribution of ion channels and morphologic properties. Post-SE, we observed this difference in the intrinsic properties was absent, yet the morphologic properties were intact. This led us to hypothesize that the distribution of ion channels had changed post-SE. Furthermore, we suspected that the ion channels, previously shown to have a dorsoventral gradient under normal conditions would be the most likely candidates to be responsible for this change. To test this hypothesis, we evaluated the dorsoventral expression of three channel types M, GIRK, and HCN in the post-SE model of TLE.

### M (K_v_7) channel expression is unchanged in epilepsy model

**Figure 8.**
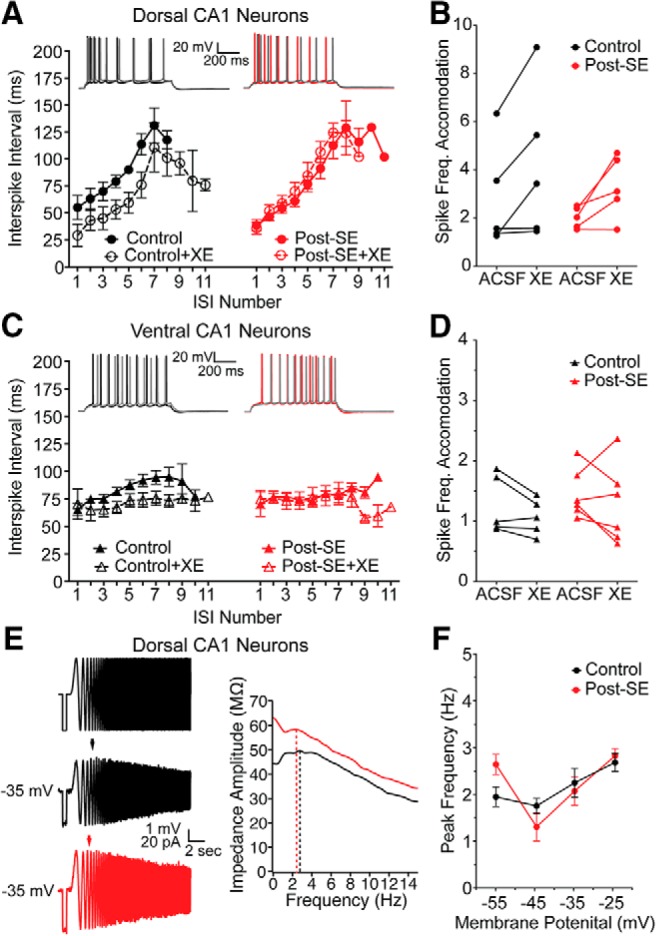
Reduced ISI in dorsal CA1 neurons post-SE cannot be explained by a reduction in M channel expression. *A–D*, Action potential trains containing 8–11 spikes were analyzed as in Figure 4. Example spike trains evoked from –60 mV are shown before and after (gray) 10 µM XE991. Summary graph shows ISI duration versus position in spike train before (solid line/closed circles) and after XE991 (dashed line/open circles). ***A***, In dorsal CA1 neurons from control and post-SE rats, the duration of the ISI increases throughout the train. ***B***, The SFA, calculated as a ratio of the first to the sixth spike, was not significantly different between control and post-SE dorsal neurons; however, there was a significant effect of XE. ***C***, In ventral CA1 neurons from control and post-SE rats, the duration of the ISI remains at ∼100 ms throughout the train. There are no differences between control and post-SE conditions. ***D***, The SFA was close to 1, and XE991 had a minimal effect on these neurons. There was no difference between control and post-SE ventral neurons. ***E***, Representative traces to the left are, from top to bottom, of injected current (–50 pA, 500-ms step and a ±50 pA, 1- to 15-Hz chirp stimulus), and representative voltage responses for dorsal CA1 neurons from control and post-SE groups at –35 mV. Arrow shows peak resonance frequency. To the right, an overlay of the impedance amplitude profiles from the representative traces with dotted lines representing the peak resonance frequency. ***F***, Peak resonance frequency at depolarized membrane potentials is graphed. Control and post-SE dorsal neurons were not different.

We first queried the expression of ion channels shown under normal conditions to be enriched in dorsal CA1 neurons hypothesizing that the development of epilepsy might cause a dorsoventral uniformity of expression. We hypothesized that the reduction in ISI duration observed in dorsal CA1 neurons post-SE could be caused by a reduction of M current (Fig. 4). To test this, we compared two M-dependent membrane properties: pharmacological sensitivity of the ISI and resonance at depolarized membrane potentials. Dorsal CA1 neurons were held slightly depolarized, at –60 mV, to increase the probability of M channel opening, and evoked action potentials were measured from the soma. Measurements were taken before (ACSF) and in the presence of 10 µM XE991, an M channel blocker. Consistent with our previous observations, we saw a mid-train reduction in the ISI post-SE (control: *n* = 5 cells/*N* = 5 rats, post-SE: *n* = 5 cells/*N* = 5 rats; multiple *t* tests using Holm–Sidak correction, *p* = 0.02 ISI 6 and 7; Figs. 4*F*, 8*A*). We compared the spike frequency adaption (SFA) between control and post-SE groups, and found that while XE-991 caused a significant reduction in SFA, there was no difference between the treatment groups (RM two-way ANOVA, *F*_(1,8)_ = 0.4707, *p* = 0.51), however there was a significant effect of XE-991 (RM two-way ANOVA *F*_(1,8)_ = 14.07, p=.01; Fig. 8*B*). In ventral CA1 neurons, which do not accommodate like dorsal CA1 neurons, bath application of XE991 had no effect on the ISI (control: *n* = 4 cells/*N* = 4 rats; post-SE: *n* = 4 cells/*N* = 4 rats; multiple *t* tests using Holm–Sidak correction, *p* > 0.05 for all ISIs; Fig. 8*C*), and there was no difference in the SFA between control and post-SE groups (RM two-way ANOVA, *F*_(1,9)_ = 2.65, *p* = 0.14; Fig. 8*D*). These results are consistent with the differences in XE991-sensitivity in dorsal and ventral neurons reported by Hönigsperger et al. (2015).

In our second test of M-dependent membrane properties, we measured resonance at depolarized membrane potentials. At depolarized membrane potentials, where HCN channels are not active, resonance is the result of an interaction between M channels and the membrane in CA1 neurons (Hu et al., 2002). We compared the peak resonance frequency at membrane potentials from –55 to –25 mV in dorsal neurons from control and post-SE animals. We did not see any difference in the peak frequency at these voltages between control and post-SE dorsal neurons (control: *n* = 6 cells/*N* = 4 rats, post-SE: *n* = 4 cells/*N* = 3 rats; RM two-way ANOVA *F*_(3,30)_ = 2.60), *p* = 0.07; Fig. 8*E*,*F*).

**Figure 9.**
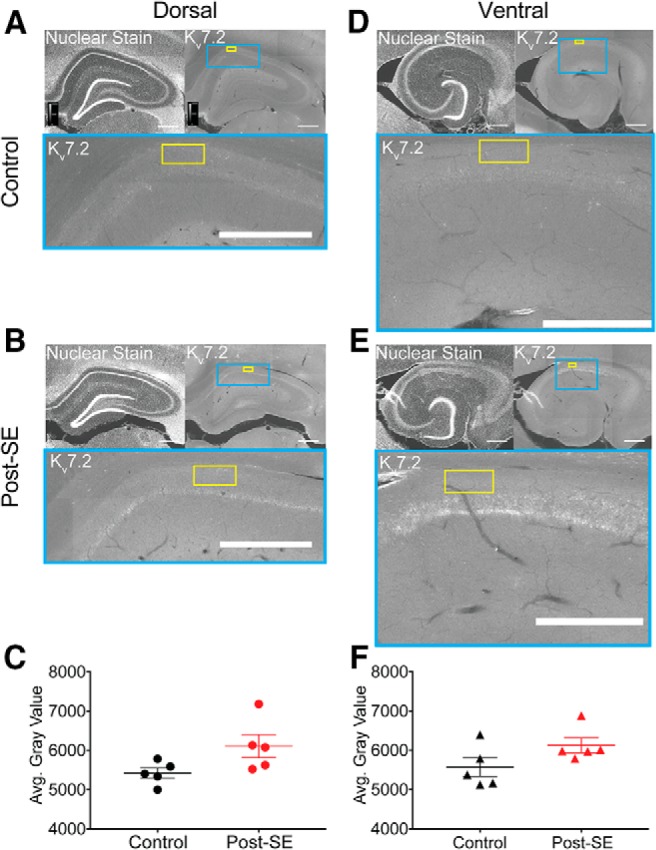
Kv7.2 immunoreactivity does not change post-SE. ***A***, ***B***, ***D***, ***E***, All representative images follow the same format. Upper left, A transverse hippocampal section with a nuclear stain provides local histologic landmarks. Upper right, Kv7.2 staining in the same slice. The blue box shows the region of CA1 expanded below. The yellow box represents the quantification area. Bottom, Zoomed in view of CA1 with subunit stain and overlay of quantification area. Scale bars = 500 µm. ***A***, Representative section from the dorsal hippocampus with Kv7.2 staining from a control rat. ***B***, Representative section from the dorsal hippocampus with Kv7.2 staining from a post-SE rat. ***C***, Group data of the mean gray value of region in stratum oriens of dorsal hippocampal slices from control and post-SE groups. These data are not statistically different. ***D***, Kv7.2 staining in the ventral hippocampus of a control rat. ***E***, Kv7.2 staining in the ventral hippocampus of a post-SE rat. ***F***, Summary data showing the average gray value in control and post-SE groups. These data are not statistically different from one another.

We also examined the relative protein staining of K_v_7.2, a commonly expressed M channel subunit which is linked to the genetic epilepsy, benign familial neonatal convulsions (Biervert et al., 1998; Singh et al., 1998). Dorsal and ventral slices from control and post-SE groups were immunolabeled and imaged (Fig. 9). We targeted the stratum oriens for quantification, since the highest expression of M channels is in the axon (Devaux et al., 2004; Pan et al., 2006). A rectangular region of interest (schematized in yellow) of equal size was overlaid on each slice and the gray value of each pixel was averaged to compute the mean gray value (Fig. 9*A*,*B*,*D*,*E*). We did not find an epilepsy-induced difference in expression in either dorsal (control: 5421 ± 131.2 A.U., *n* = 2 sections/*N* = 5 animals, post-SE: 6107 ± 294.5 A.U., *n* = 2 sections/*N* = 5 animals; unpaired *t* test, *p* = 0.07; Fig. 9*C*) or ventral CA1 (control: 5570 ± 237.7 A.U., *n* = 2 sections/*N* = 5 animals, post-SE: 6127 ± 193.4 A.U., *n* = 2 sections/*N* = 5 animals; unpaired *t* test, *p* = 0.11; Fig. 9*F*). One caveat with this method of quantification is that we were unable to distinguish K_v_7.2 labeling of CA1 pyramidal neurons from other axons in the region such as interneurons and Schaffer collaterals. In sum, our analysis of M channel expression using physiological and biochemical approaches suggested that there is no difference between control and post-SE expression levels.

### Epileptic network activity did not induce GIRK channel plasticity

**Figure 10.**
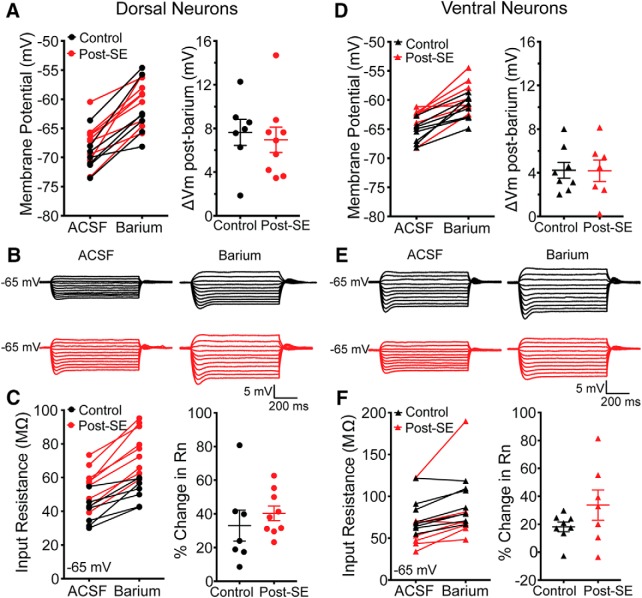
The functional expression of GIRK channels is unaltered in dorsal and ventral neurons post-SE. ***A***, With the bath application of 50 µM barium, the membrane potential increased in both control and post-SE groups. The barium induced depolarization was 7.6 ± 1.2 mV in controls and 7.0 ± 1.2 mV in the post-SE group. This difference was not statistically significant (unpaired *t* test, *p* = 0.69). ***B***, Representative voltage traces held at –65 mV from which the input resistance was calculated in ACSF (baseline) and barium conditions in cells from control and post-SE groups. ***C***, Somatic input resistance at –65 mV before and after application of barium in control and post-SE. Relative to baseline, the input resistance in controls increased by 33.0 ± 9.2%, and post-SE, the increase was 40.3 ± 4.4%. The change in input resistance was not statically different between the two groups. ***D***, Bath application of 50 µM barium caused the membrane potential of ventral CA1 neurons from both groups to depolarize. Summary graphs show the change in the control and post-SE groups. The change in membrane potential was not different between the two groups. ***E***, Representative voltage traces held at –65 mV from which the input resistance was calculated in ACSF (baseline) and barium conditions in control (black) and post-SE (red). ***F***, Barium caused the steady state input resistance to increase in ventral CA1 neurons from both control and post-SE groups. The percentage change relative to baseline was 18.1 ± 3.5% for controls and 33.7 ± 10.8% post-SE. This difference was not statistically significant.

We hypothesized that a reduction in GIRK channel expression, might contribute to the increase in input resistance we observed in dorsal neurons post-SE. To test whether GIRK channel expression was altered post-SE, we bath applied a low concentration of barium (50 µM) to block inward rectifiers (Kim and Johnston, 2015). We compared the effect of barium on the membrane potential and input resistance within the dorsal and ventral regions. When recording from the soma of dorsal neurons, barium caused a prominent depolarization of ∼7 mV in both control and post-SE groups (control: 7.6 ± 1.2 mV, *n* = 7 cells/*N* = 5 rats, post-SE: 7.0 ± 1.2 mV, *n* = 9 cells/*N* = 5 rats; unpaired *t* test, *p* = 0.69; Fig. 10*A*). Barium increased the input resistance in dorsal CA1 neurons from both control and post-SE groups (Fig. 10*B*,*C*). We saw an average increase of 33 ± 9.2% in control neurons (*n* = 7 cells/*N* = 5 rats) and 40.3 ± 4.4% post-SE (*n* = 9 cells/*N* = 5 rats). When statistically compared, we could not distinguish these two groups (unpaired *t* test, *p* = 0.45). We saw a similar pattern in ventral CA1 neurons. Barium caused an increase of 4 mV in the resting membrane potential in both control and post-SE neurons (control: 4.22 ± 0.73 mV *n* = 8 cells/*N* = 7 rats, post-SE: 4.18 ± 0.98 mV *n* = 7 cells/*N* = 5 rats; unpaired *t* test, *p* = 0.69; Fig. 10*D*). The input resistance increased with barium (control: 18.1 ± 3.5% *n* = 8 cells/*N* = 7 rats, post-SE: 33.7 ± 10.8% *n* = 7 cells/*N* = 5 rats), but the two groups were not different from one another (unpaired *t* test, *p* = 0.17; Fig. 10*E*,*F*).

**Figure 11.**
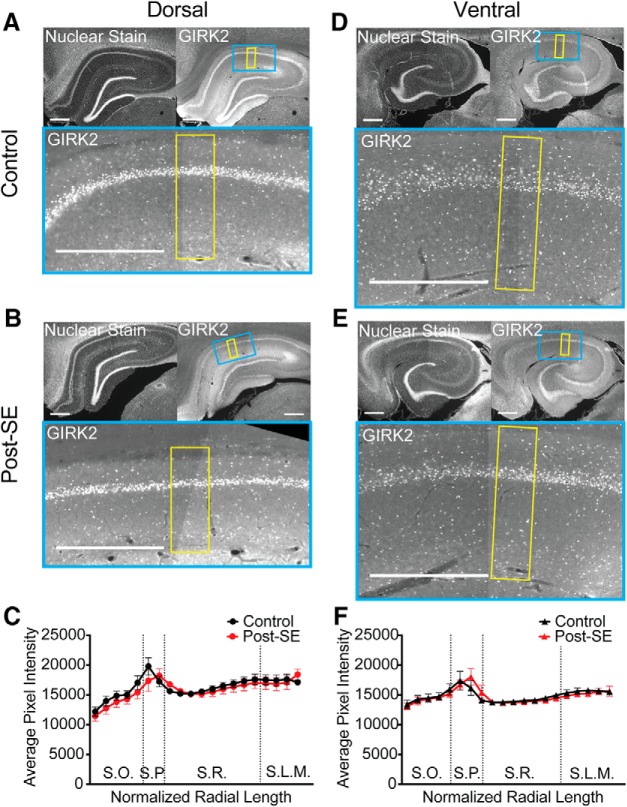
Expression of GIRK2 subunit is unchanged post-SE. ***A***, ***B***, ***D***, ***E***, All representative images follow the same format. Upper left, Transverse slice from dorsal hippocampus with the nuclear stain, Hoechst 33342, from control group. Upper right, Representative hippocampal staining of GIRK2. The blue box shows the portion of CA1 expanded below. The yellow shaded region shows the region selected for quantification from the alveus to the fissure in both channels. Bottom, GIRK2 staining in CA1, where the lighter shade of gray reflects more immunoreactivity for GIRK2 protein. Staining is evident in the somatic layer (S.P.) and dendritic layers. Scale bars = 500 µm. ***A***, Representative section from the dorsal hippocampus with GIRK2 staining from a control rat. ***B***, Representative section from the dorsal hippocampus with GIRK2 staining from a post-SE rat. ***C***, Quantification of average grayscale pixel intensity along the length of the somatodendritc axis on dorsal CA1. Since the radial length can differ between sections, the lengths were normalized and binned into 20 segments. Dotted lines reflect transitions between layers abbreviated S.O. (stratum oriens), S.P. (stratum pyramidale), S.R. (stratum radiatum), and S.L.M. (stratum lacunosum moleculare). Comparisons between equivalent radial locations were tested between control and post-SE group data. ***D***, GIRK2 staining in the ventral hippocampus of control rat. ***E***, GIRK2 staining in the ventral hippocampus of a post-SE rat. ***F***, Quantification along the normalized length of the somatodendritic/radial axis in ventral CA1. Equivalent radial locations were compared between control and post-SE group data.

Girk2 (Kir3.2) is one of the most ubiquitously expressed subunits of GIRK channels in the hippocampus (Karschin et al., 1996; Liao et al., 1996). The absence of GIRK2 causes spontaneous seizures in mice (Signorini et al., 1997). Representative images show a nuclear stain in the upper left and GIRK2 staining in the upper right with an expanded view of CA1 below (Fig. 11*A*,*B*,*D*,*E*). In agreement with the results from the whole-cell recordings, we did not see an epilepsy induced difference in GIRK2 protein expression in either the dorsal (*n* = 2 slices/*N* = 5 animals, multiple *t* test with Holm–Sidak correction, *p* = 0.99; Fig. 11*C*) or ventral hippocampus (*n* = 2 slices/*N* = 5 animals, multiple *t* test with Holm–Sidak correction, *p* = 0.99; Fig. 11*F*).

In summary, we had hypothesized that a reduction in GIRK channel expression could explain the increased input resistance of dorsal CA1 neurons post-SE. Our data, however, did not support this hypothesis. Instead, it appears GIRK expression is resistant to seizure induced plasticity in this model.

### HCN channel expression is reduced in dorsal CA1 neurons post-SE

**Figure 12.**
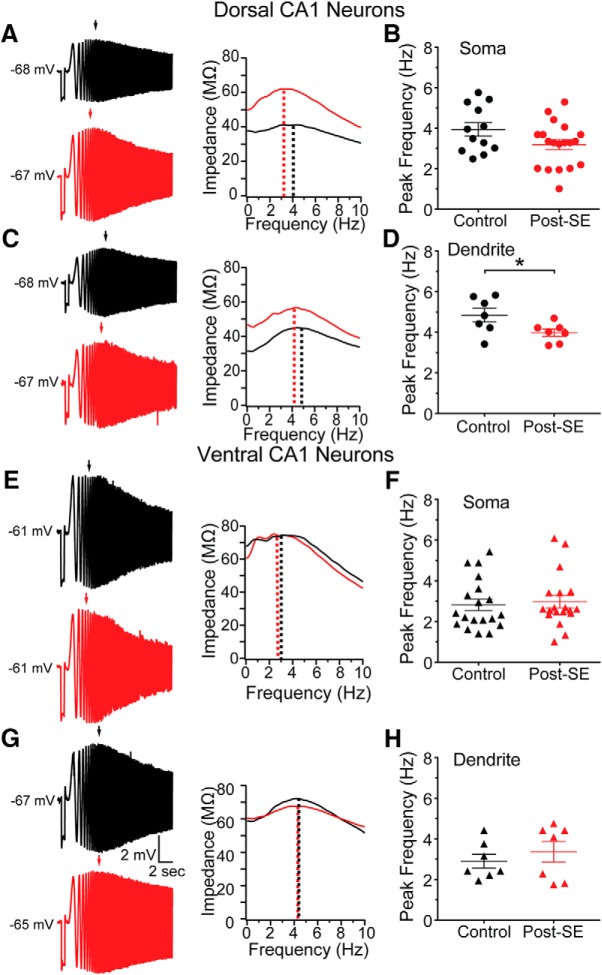
Resonance frequency is reduced in dorsal dendrites post-SE. ***A***, ***C***, ***E***, ***G***, left, Representative voltage responses to a 15-Hz chirp stimulus obtained from whole-cell recordings from control (upper, black) and post-SE (lower, red) cells. Right, Plot of impedance amplitude as a function of frequency for voltage traces. Dotted line shows the peak frequency. ***A***, Representative recordings from the soma of dorsal CA1 neurons. ***B***, Summary graph showing the peak resonance frequency values obtained from dorsal somatic recordings from control and post-SE groups. These groups were not statically different from one another. ***C***, Representative recordings from the apical dendrite of dorsal CA1 neurons. ***D***, Summary of data collected from the dendrite of dorsal CA1 neurons showing the peak resonance in control and post-SE groups. These groups were statistically different. ***E***, Representative responses at the soma of ventral CA1 neurons in both groups. ***F***, Summary graphs showing the group data for control and post-SE conditions. ***G***, Representative recordings at the dendrite of ventral CA1 neurons. ***H***, Summary graphs comparing the group data for control and post-SE conditions. *p < 0.05.

We hypothesized a reduction in the expression of HCN channels could contribute to the increased input resistance of dorsal CA1 neurons post-SE. To test this, we measured (1) resonance frequency, an intrinsic property associated with HCN channel expression and (2) sensitivity to the blocker, ZD7288. In CA1 neurons at subthreshold membrane potentials, resonance is primarily an interplay between the passive properties of the membrane and HCN channels (Narayanan and Johnston, 2007). In our recordings from the soma of dorsal neurons, the peak resonance frequency was not significantly different between control and post-SE neurons (control: 3.93 ± 0.33 Hz, *n* = 12 cells/*N* = 9 rats, post-SE: 3.18 ± 0.25 Hz, *n* = 19 cells/*N* = 14 rats; unpaired *t* test, *p* = 0.08; Fig. 12*A*,*B*). In the dendrite of dorsal CA1 neurons, however, we found that the peak resonance frequency post-SE was reduced by ∼1 Hz compared to control (control: 4.84 ± 0.34 Hz, *n* = 7 dendrites/*N* = 6 rats, post-SE: 3.98 ± 0.19 Hz, *n* = 7 dendrites/*N* = 7 rats; unpaired *t* test, *p* = 0.04; Fig. 12*C*,*D*). In ventral CA1 neurons we did not detect a difference in the peak resonance frequency at the soma (control: 2.86 ± 0.29 Hz, *n* = 19 cells/*N* = 15 rats, post-SE: 2.98 ± 0.30 Hz, *n* = 19 cells/*N* = 14 rats; unpaired *t* test, *p* = 0.72; Fig. 12*E*,*F*) or dendrite post-SE (control: 2.90 ± 0.34 Hz, *n* = 7 dendrites/*N* = 7 rats, post-SE: 3.36 ± 0.51 Hz, *n* = 7 dendrites/*N* = 7 rats; unpaired *t* test, *p* = 0.47; Fig. 12*G*,*H*). HCN channels have been shown to play a more prominent role in the subthreshold properties of ventral CA1 neurons, due to a right-shifted voltage dependence (Dougherty et al., 2013). Therefore, it is surprising that we did not detect a change in peak resonance frequency in ventral CA1 neurons post-SE. While the intrinsic excitability appeared to become more uniform across the dorsoventral axis, these data would suggest that the gradient of HCN expression is more pronounced post-SE.

**Figure 13.**
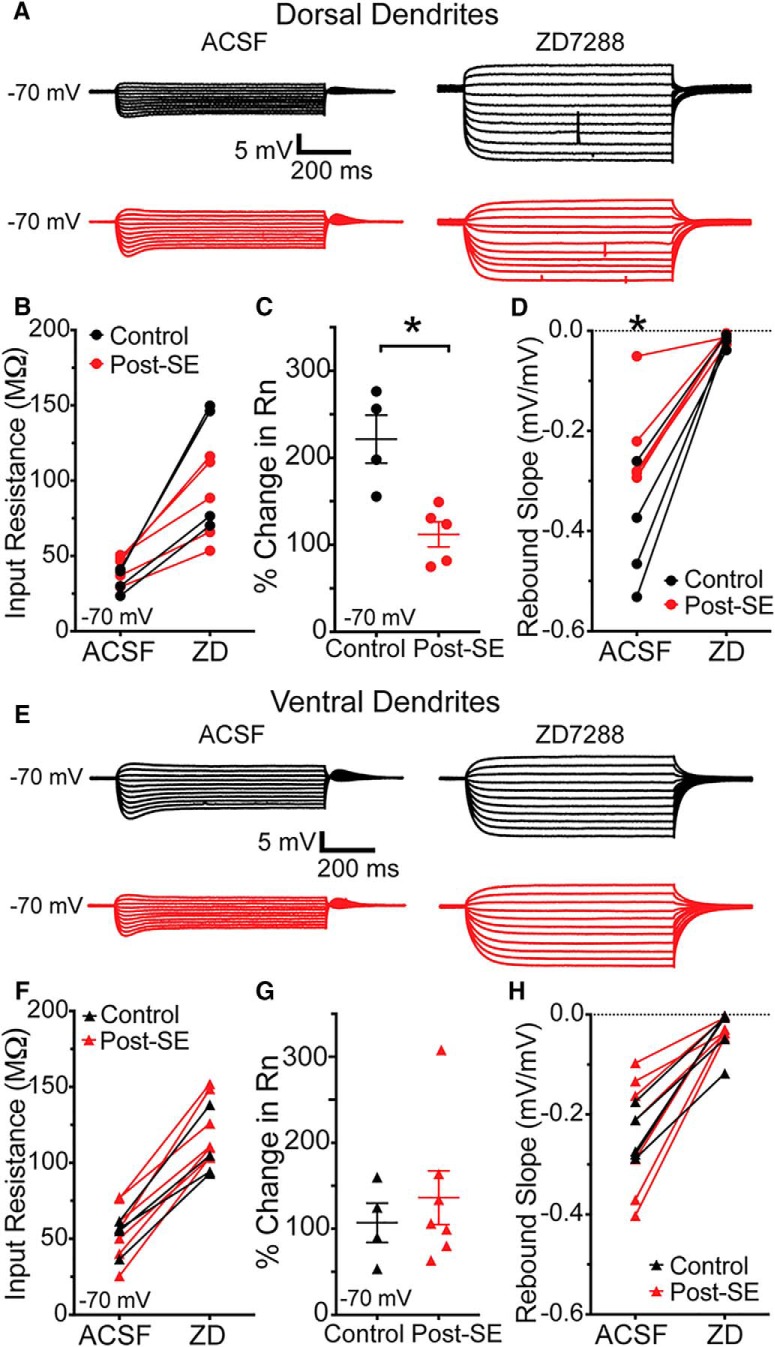
Reduced sensitivity to the HCN channel blocker, ZD7288, in dorsal dendrites post-SE. ***A***, Voltage responses at the dendrite of dorsal CA1 neurons were measured from –70 mV under baseline conditions, ACSF, and after bath application of 10 µM ZD7288. ***B***, The steady state input resistance increased after ZD was introduced in both control and post-SE groups. ***C***, The increase in input resistance was much larger in controls. ***D***, The amplitude of the rebound depolarization was plotted as a function of the membrane potential at the end of the current step. The slope of this relationship was plotted under baseline, ACSF, conditions and then after ZD7288 for both treatment groups. In dorsal CA1 neurons post-SE, the rebound slope was significantly reduced. ***E***, Representative traces from dendrites of ventral CA1 neurons under baseline conditions, ACSF, or after bath application of 10 µM ZD7288. ***F***, The dendritic input resistance of ventral CA1 neurons increased after ZD7288. ***G***, The increase in input resistance relative to baseline was not different between the groups. ***H***, The rebound slope was reduced with application of ZD, but there was no difference between the control and post-SE groups. *p < 0.05.

We then assessed the effect of 10 µM ZD7288, an HCN channel blocker, on the input resistance and rebound slope at the dendrite of dorsal and ventral CA1 neurons post-SE. A larger change in input resistance is indicative of an increase in functional HCN channels. Similarly, the rebound slope is an indirect measured of HCN channels, where a more negative slope signifies more I_h_. At –70 mV, a membrane potential where HCN channels would normally be active, ZD7288 caused the dendritic steady state input resistance to increase in dorsal CA1 neurons from control rats (ACSF: 33.6 ± 4.2 MΩ, ZD7288: 110.7 ± 21.6 MΩ; *n* = 4 dendrites/*N* = 4 rats; Fig. 13*A*,*B*). The dendritic input resistance of dorsal CA1 neurons post-SE increased, but to a lesser extent than controls (ACSF: 42.5 ± 4.0 MΩ, ZD7288: 87.3 ± 12.4 MΩ, *n* = 5 dendrites/*N* = 5 rats; Fig. 13*A*,*B*). The relative change in input resistance was significantly reduced in the post-SE group (control: 221.5 ± 27.6%, post-SE: 112.1 ± 14.4%, unpaired *t* test, *p* = 0.01; Fig. 13*C*). Before ZD7288 bath application, the rebound slope at the dendrite of dorsal CA1 neurons was reduced post-SE compared to control (control: –0.41 ± 0.06 mV/mV, *n* = 4 dendrites/*N* = 4 rats, post-SE: –0.23 ± 0.05 mV/mV, *n* = 5 dendrites/*n* = 5 rats, two-way ANOVA *F*_(1,7)_ = 5.69, Sidak *post hoc*, *p* = 0.01; Fig. 13*D*). This reduction, like the smaller change in input resistance, is consistent with less I_h_. Following application of ZD7288, the dendritic input resistance of ventral CA1 neurons increased in both control (ACSF 52.5 ± 5.5 MΩ, ZD7288 107.5 ± 10.6 MΩ, *n* = 4 dendrites/*N* = 4 rats) and post-SE (ACSF 55.3 ± 7.1 MΩ, ZD7288 122.0 ± 7.8 MΩ, *n* = 7 dendrites/*n* = 6 rats) groups. The relative change, however was not different between the two groups (control: 107.0 ± 22.7%, *n* = 4 dendrites/*N* = 4 rats, post-SE: 136.2 ± 31.3%, *n* = 7 dendrites/*N* = 6 rats, unpaired *t* test, *p* = 0.54; Fig. 13*E–G*). Similarly, the rebound slope was not different between control and post-SE groups in ventral CA1 neurons (RM two-way ANOVA, *F*_(1,10)_ = 0.01, *p* = 0.93; Fig. 13*H*).

**Figure 14.**
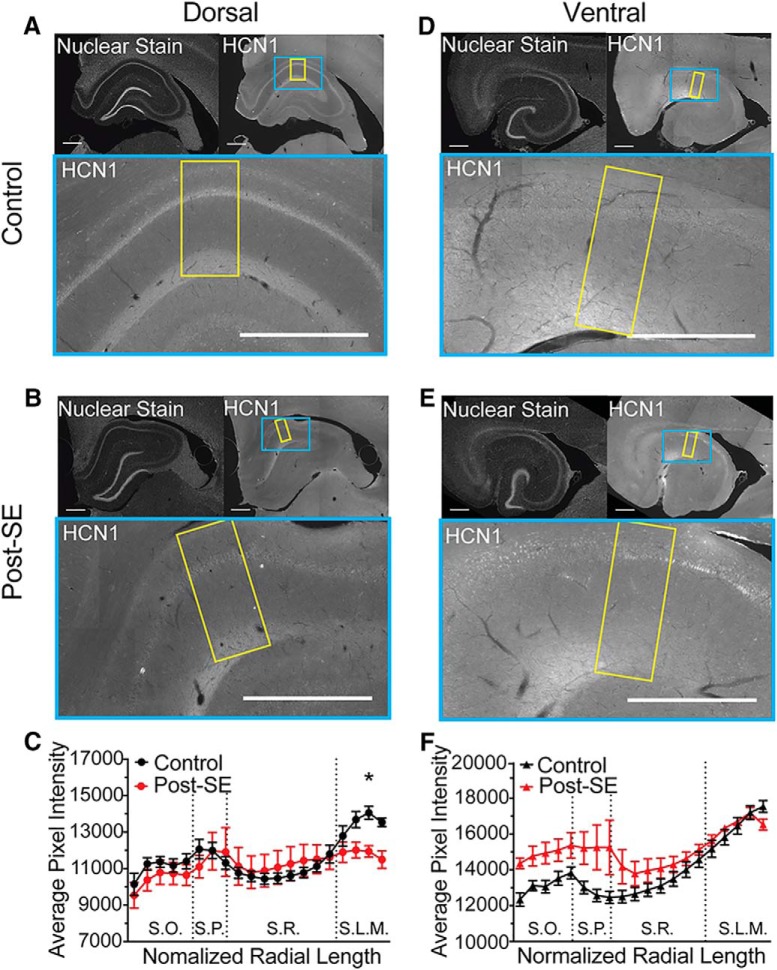
Reduced expression of HCN1 subunit in distal dendritic layer post-SE. ***A***, ***B***, ***D***, ***E***, Upper left, Representative hippocampal section with the nuclear stain. Upper right, HCN1 staining. The blue box shows the portion of CA1 expanded below. The yellow box shows the region selected for quantification from the alveus to the fissure. Bottom, HNC1 staining in CA1, where the lighter shade of gray reflects more immunoreactivity for HCN1 protein. The most prominent staining is in the distal dendrites of S.L.M. Scale bars = 500 µm. ***B***, Representative section shown of dorsal hippocampal section from post-SE rat. ***C***, Quantification along the length of the somatodendritc axis on dorsal CA1. Dotted lines reflect transitions between layers. Comparisons between equivalent radial locations were tested between control and post-SE group data. ***D***, HCN1 staining in the ventral hippocampus of a control rat. ***E***, HCN1 staining in the ventral hippocampus of a post-SE rat. ***F***, Quantification along the normalized length of the somatodendritic axis in ventral CA1. Equivalent radial locations were compared between control and post-SE group data. *p < 0.05.

A reduction in resonance frequency and sensitivity to ZD could be caused by a reduction in channel expression or an alteration in the biophysical properties of channels in the membrane. To test whether the expression pattern of HCN channels changed post-SE, we measured the immunoreactivity to the HCN1 channel subunits in dorsal and ventral CA1. For each panel, the upper left inset shows the nuclear staining pattern for each section revealing the gross hippocampal histology (Fig. 14*A*,*B*,*D*,*E*). In the expansion of CA1 below the HCN1 immunofluorescence is shown. Under control conditions, we saw an increase in HCN1 staining in the distal dendritic layer of SLM of both dorsal and ventral CA1 (Fig. 14*A*,*D*). In the dorsal CA1 post-SE, this increase in HCN1 labeling in the distal dendrites was absent; the level of staining in SLM was equivalent to stratum radiatum (SR; *n* = 2 slices/*N* = 5 animals; multiple *t* test with Holm–Sidak correction, bin 19, *p* = 0.038; Fig. 14*C*). In ventral CA1, we found that in both control and post-SE sections the HCN1 staining increased with distance from the soma (*n* = 2 slices/*N* = 5 animals; multiple *t* test with Holm–Sidak correction, *p* = 0.53–0.97; Fig. 14*D–F*). In one post-SE animal, which was included in the analysis, there was an increase in brightness in the pyramidal layer (SP) and oriens (SO), which we believe was due to damage rather than an increase in perisomatic HCN1 staining, since it was present in the absence of the primary antibody. Both the physiological recordings and immunohistochemistry suggest that post-SE, the expression of HCN channels is reduced in the dendrites of dorsal CA1 neurons, while the HCN expression in ventral CA1 neurons is unchanged.

## Discussion

In this study, we have investigated how the electrical properties of CA1 neurons are altered as the hippocampal network becomes permissible to spontaneous seizures using a post-SE model of TLE. Previous research suggests epilepsy causes the input-output relationship in CA1 neurons to change, which results in an increased intrinsic excitability (Ketelaars et al., 2001; Su et al., 2002; Bernard et al., 2004; Jung et al., 2007; Oliveira et al., 2010). We tested whether this increased excitability uniformly affects neurons at either end of the longitudinal axis of the hippocampus. We found that the intrinsic excitability is increased in dorsal CA1 neurons, but not ventral CA1 neurons post-SE. Dorsal CA1 neurons post-SE had an increased firing intensity that was accompanied by a reduced ISI and increased input resistance at the soma and apical dendrite. The intrinsic properties of neurons are determined by neuronal morphology and ion channel distribution. We ruled out epilepsy induced changes in morphology with cellular reconstructions. Therefore, we tested for disruptions in ion channel expression that were specific to dorsal CA1 neurons post-SE. To explain the reduction in ISI we measured, we tested for a reduction in M/K_v_7 channel expression. We did not detect a difference in the pharmacological sensitivity of the ISI or in the expression of the Kv7.2 subunit post-SE. To explain the increased input resistance at the soma and dendrite post-SE, we tested for a reduction in GIRK and HCN channel expression. We did not detect a difference in the pharmacological sensitivity of GIRK-dependent intrinsic properties or in the expression of the GIRK2 channel subunit between control and post-SE conditions. At the dendrites of dorsal CA1 neurons post-SE we saw a reduction in the ZD-7288-sensitive component of the input resistance suggesting there is less I_h_ post-SE. We also found a reduction in the HCN1 immunostaining in the dorsal dendrites post-SE. This reduction in HCN expression in dorsal, but not ventral CA1 neurons, contributed to the increased intrinsic excitability in dorsal CA1 neurons. This difference between the phenotypes of dorsal and ventral CA1 neurons post-SE was surprising and suggested that epilepsy does not uniformly affect CA1 neurons.

### Differences between the excitability of dorsal and ventral CA1 neurons is absent post-SE

At the inception of this study we hypothesized that epilepsy would cause a uniform increase in excitability on top of the normal gradient. In this case, dorsal-ventral differences in excitability would still be evident, and all CA1 neurons post-SE would be more likely to engage in epileptiform activity. While this was a compelling hypothesis, it was not supported by the data. A main finding in this study was that in this model of epilepsy dorsal but not ventral neurons become more excitable (Figs. 3–6).

It is surprising that we observed epilepsy-induced changes exclusively in dorsal CA1 neurons, because the ventral hippocampus is more tightly associated with seizure generation and seizure induced damage (Elul, 1964; Racine et al., 1977; Gilbert et al., 1985; Bragdon et al., 1986; Cavazos et al., 2004; Ekstrand et al., 2011; Toyoda et al., 2013). Our results indicated that the excitability of dorsal CA1 neurons post-SE increased and became indistinguishable from ventral CA1 neurons. This change may permit dorsal neurons to function more like ventral neurons, extending the excitability phenotype of ventral neurons across the dorsoventral axis of CA1 post-SE.

The dorsal and ventral hippocampus are associated with different aspects of behavior. Lesion studies have shown the dorsal, but not the ventral, hippocampus is necessary for spatial navigation (Moser et al., 1995). Other lesion studies have implicated the ventral hippocampus in the encoding of emotional aspect of experience, including fear and autonomic processes (Bannerman et al., 2002; Kjelstrup et al., 2002). Our data would suggest that the behavioral role of the ventral hippocampus has not changed in epilepsy, but that spatial navigation requiring the dorsal hippocampus could be adversely affected. Place cells in the dorsal hippocampus have, in fact, been found to be less stable and precise post-SE (Liu et al., 2003).

### HCN channelopathies in TLE

One interesting juxtaposition is that while the excitability of dorsal and ventral neurons became more similar post-SE, there was a greater difference in the expression of HCN channels between dorsal and ventral regions. In 2013, Dougherty and colleagues showed in naïve rats ventral CA1 neurons have an increase in the ratio of HCN1 to HCN2 subunits compared to dorsal neurons, which caused HCN channels to play a more prominent role in the resting properties of ventral CA1 neurons (Dougherty et al., 2013). Here, we show HCN channel expression decreased in dorsal CA1 neurons, contributing to the increased intrinsic excitability observed in dorsal CA1 neurons.

These data add to the literature linking HCN channels with epilepsy models. One compelling hypothesis is that in epilepsy dentate granule cells reflect homeostatic ion channel plasticity, while CA1 pyramidal neurons exhibit acquired channelopathies (Wolfart and Laker, 2015). Consistent with this framework, HCN channel expression is increased in granule cells, reducing the excitability of these neurons (Bender et al., 2003). In CA1 neurons, however, reduced HCN channel expression (mRNA, total protein, and surface labeling) has been found in epilepsy models (Jung et al., 2007; Powell et al., 2008; Shin et al., 2008; Huang et al., 2009; Marcelin et al., 2009; Santoro et al., 2010; Jung et al., 2011). Reduced I_h_ has also been found in recordings from human layer II/III pyramidal neurons in the entorhinal cortex (Wierschke et al., 2010), a cell type that, like CA1 pyramidal neurons, had a reduction in I_h_ post-SE (Shah et al., 2004). Furthermore, modulation of I_h_ has been associated with seizure susceptibility. For example, mice without the HCN1 protein have an increased susceptibility to seizures (Huang et al., 2009; Santoro et al., 2010). In addition, preventing the transcriptional repression of the HCN1 gene in the dorsal hippocampus by the nonspecific transcriptional repressor, NRSF, decreased the number of spontaneous seizures observed one to two weeks post-SE (McClelland et al., 2011).

HCN channel have been suggested as a potential target for the treatment of epilepsy (Chen et al., 2002; Shah et al., 2013; Noam et al., 2011). Some currently available antiseizure drugs (ASDs) have been shown to increase I_h_ in CA1 neurons [e.g., gabapentin (Surges et al., 2003) and lamotrigine (Poolos et al., 2002)]. Therefore, our study suggests that the therapeutic effect of these drugs in CA1 neurons may be different in dorsal and ventral CA1 neurons. More specifically, the ASD-induced increase in I_h_ could serve to rectify a disease phenotype in the dorsal region of CA1, while causing a homeostatic reduction in excitability in the ventral CA1.

Several potential mechanisms could cause a reduction in HCN channels. Disruptions in both the transcription and post-translational regulation of HCN subunits post-SE has been found (Jung et al., 2010; McClelland et al., 2011; Williams et al., 2015). In addition, the assembled subunit composition and trafficking of channels is sensitive to seizures, particularly the HCN1 subunit (Shin et al., 2008; Zha et al., 2008; Marcelin et al., 2009).

HCN channels conduct an inward, cationic current when the membrane potential hyperpolarizes, which reverses around –40 mV, close to the action potential threshold. The effect of this current is to limit the spatial and temporal summation of excitatory inputs and the channel kinetics bias the propagation of inputs that arrive at theta frequencies, 4–12 Hz (Magee, 1999; Narayanan and Johnston, 2007; Vaidya and Johnston, 2013). A reduction in the dendritic HCN channel expression not only increases the time window for summation, but also disrupts the integration of tuned inputs (Magee, 1999; Nolan et al., 2004). The strength of inputs, especially those from the temporoammonic pathway have been shown to be enhanced post-SE (Wozny et al., 2005; Ang et al., 2006). Similarly, a loss of I_h_ in dorsal CA1 neurons, and O-LM interneurons has been correlated with a reduction in intracellular and extracellular theta oscillations (Buzsáki, 2002; Dugladze et al., 2007; Marcelin et al., 2009). These data suggest that the loss of I_h_ is detrimental to both neuronal and circuit level function.

### Incorporating longitudinal position into future hippocampal studies

The awareness of differences in HCN expression along the dorsovental axis was recently reported (Marcelin et al., 2012a; Dougherty et al., 2013). Identifying the longitudinal position of experiments done in CA1 before these studies is not feasible. We were, however, able to recover a subset of the slices used for the physiological recordings reported by Shin et al. (2008). The acute slices were fixed with gluteraldehyde immediately after the recordings were made and we measured the anatomic properties using the algorithm developed by Malik et al. (2016). The longitudinal position of twenty-one slices used in their experiments had a mid-point of –1.27 ± 0.15 mm, which would be classified as ventral-intermediate. Therefore, we hypothesize that HCN channel expression is reduced along three quarters of the longitudinal axis of CA1 (from dorsal to ventral-intermediate). The reduction in HCN channel expression we report here compliments the existing literature by providing an anatomic context in which these changes occur. Future investigation will be needed to determine if other acquired channelopathies associated with TLE also have a differential expression pattern along the dorsoventral axis of the hippocampus.

## Synthesis

Reviewing Editor: Katalin Toth, Universite Laval Decisions are customarily a result of the Reviewing Editor and the peer reviewers coming together and discussing their recommendations until a consensus is reached. When revisions are invited, a fact-based synthesis statement explaining their decision and outlining what is needed to prepare a revision will be listed below. Note: If this manuscript was transferred from JNeurosci and a decision was made to accept the manuscript without peer review, a brief statement to this effect will instead be what is listed below.

The study provides important new information about cellular pathophysiological changes in epilepsy. It is built on high quality data, meticulously analyzed and technically very solid. The authors have addressed the reviewer's concerns raised at the previous review cycle.

## References

[B1] Amaral DG, Witter MP (1989) The three-dimensional organization of the hippocampal formation: a review of anatomical data. Neuroscience 31:571–591. 2687721 10.1016/0306-4522(89)90424-7

[B2] Ang CW, Carlson GC, Coulter DA (2006) Massive and specific dysregulation of direct cortical input to the hippocampus in temporal lobe epilepsy. J Neurosci 26:11850–11856. 10.1523/JNEUROSCI.2354-06.2006 17108158 PMC2175390

[B3] Aronica E, Boer K, Doorn KJ, Zurolo E, Spliet WGM, van Rijen PC, Baayen JC, Gorter JA, Jeromin A (2009) Expression and localization of voltage dependent potassium channel Kv4.2 in epilepsy associated focal lesions. Neurobiol Dis 36:81–95. 10.1016/j.nbd.2009.06.016 19596445

[B4] Bannerman DM, Deacon RMJ, Offen S, Friswell J, Grubb M, Rawlins JNP (2002) Double dissociation of function within the hippocampus: spatial memory and hyponeophagia. Behav Neurosci 116:884–901. 10.1037/0735-7044.116.5.884 12369808

[B5] Bender RA, Soleymani SV, Brewster AL, Nguyen ST, Beck H, Mathern GW, Baram TZ (2003) Enhanced expression of a specific hyperpolarization-activated cyclic nucleotide-gated cation channel (HCN) in surviving dentate gyrus granule cells of human and experimental epileptic hippocampus. J Neurosci 23:6826–6836. 12890777 10.1523/JNEUROSCI.23-17-06826.2003 PMC3100807

[B6] Bernard C, Anderson A, Becker A, Poolos NP, Beck H, Johnston D (2004) Acquired dendritic channelopathy in temporal lobe epilepsy. Science 305:532–535. 10.1126/science.1097065 15273397

[B7] Biervert C, Schroeder BC, Kubisch C, Berkovic SF, Propping P, Jentsch TJ, Steinlein OK (1998) A potassium channel mutation in neonatal human epilepsy. Science 279:403–406. 9430594 10.1126/science.279.5349.403

[B8] Bragdon AC, Taylor DM, Wilson WA (1986) Potassium-induced epileptiform activity in area CA3 varies markedly along the septotemporal axis of the rat hippocampus. Brain Res 378:169–173. 3742197 10.1016/0006-8993(86)90300-8

[B9] Buzsáki G (2002) Theta oscillations in the hippocampus. Neuron 33:325–340. 11832222 10.1016/s0896-6273(02)00586-x

[B10] Cavazos JE, Jones SM, Cross DJ (2004) Sprouting and synaptic reorganization in the subiculum and CA1 region of the hippocampus in acute and chronic models of partial-onset epilepsy. Neuroscience 126:677–688. 10.1016/j.neuroscience.2004.04.014 15183517 PMC3179906

[B75] Chen K, Aradi I, Santhakumar V, Soltesz I (2002) H-channels in epilepsy: new targets for seizure control? Trends Pharmacol Sci. 23:552–557. 12457772 10.1016/s0165-6147(02)02110-7

[B11] Coan AC, Cendes F (2013) Epilepsy as progressive disorders: what is the evidence that can guide our clinical decisions and how can neuroimaging help? Epilepsy Behav 26:313–321. 10.1016/j.yebeh.2012.09.027 23127969

[B12] Devaux JJ, Kleopa KA, Cooper EC, Scherer SS (2004) KCNQ2 is a nodal K+ channel. J Neurosci 24:1236–1244. 10.1523/JNEUROSCI.4512-03.2004 14762142 PMC6793582

[B13] Dougherty KA, Islam T, Johnston D (2012) Intrinsic excitability of CA1 pyramidal neurones from the rat dorsal and ventral hippocampus. J Physiol 590:5707–5722. 10.1113/jphysiol.2012.242693 22988138 PMC3528986

[B14] Dougherty KA, Nicholson DA, Diaz L, Buss EW, Neuman KM, Chetkovich DM, Johnston D (2013) Differential expression of HCN subunits alters voltage-dependent gating of h-channels in CA1 pyramidal neurons from dorsal and ventral hippocampus. J Neurophysiol 109:1940–1953. 10.1152/jn.00010.2013 23324324 PMC3628004

[B15] Drakew A, Mu M, Ga BH, Thompson SM, Frotscher M (1996) Spine loss in experimental epilepsy: quantitative light and electron microscopic analysis of intracellularly stained CA3 pyramidal cells in hippocampal slice cultures. Neuroscience 70:31–45. 10.1016/0306-4522(95)00379-W 8848134

[B16] Dugladze T, Vida I, Tort AB, Gross A, Otahal J, Heinemann U, Kopell NJ, Gloveli T (2007) Impaired hippocampal rhythmogenesis in a mouse model of mesial temporal lobe epilepsy. Proc Natl Acad Sci USA 104:17530–17535. 10.1073/pnas.0708301104 17954918 PMC2077290

[B17] Ekstrand JJ, Pouliot W, Scheerlinck P, Dudek FE (2011) Lithium pilocarpine-induced status epilepticus in postnatal day 20 rats results in greater neuronal injury in ventral versus dorsal hippocampus. Neuroscience 192:699–707. 10.1016/j.neuroscience.2011.05.022 21669257 PMC3279156

[B18] Elul R (1964) Regional differences in the hippocampus of the cat. Electroencephalogr Clin Neurophysiol 16:470–488. 14159001 10.1016/0013-4694(64)90089-6

[B19] Engel J (2001) Mesial temporal lobe epilepsy: what have we learned? Neuroscientist 7:340–352. 10.1177/107385840100700410 11488399

[B20] Fanselow MS, Dong HW (2010) Are the dorsal and ventral hippocampus functionally distinct structures? Neuron 65:7–19. 10.1016/j.neuron.2009.11.031 20152109 PMC2822727

[B21] French JA (2007) Refractory epilepsy: clinical overview. Epilepsia 48 [Suppl 1]:3–7. 10.1111/j.1528-1167.2007.00992.x 17316406

[B22] Gilbert M, Racine RJ, Smith GK (1985) Epileptiform burst responses in ventral vs dorsal hippocampal slices. Brain Res 361:389–391. 4084805 10.1016/0006-8993(85)91309-5

[B23] Gowers WR (1882) Epilepsy and other convulsive diseases. J Ment Sci 28:245–259.

[B24] Heinzen EL, Yoon W, Weale ME, Sen A, Wood NW, Burke JR, Welsh-Bohmer KA, Hulette CM, Sisodiya SM, Goldstein DB (2007) Alternative ion channel splicing in mesial temporal lobe epilepsy and Alzheimer’s disease. Genome Biol 8:R32. 10.1186/gb-2007-8-3-r32 17343748 PMC1868939

[B25] Helmstaedter C, Kockelmann E (2006) Cognitive outcomes in patients with chronic temporal lobe epilepsy. Epilepsia 47:96–98. 10.1111/j.1528-1167.2006.00702.x 17105474

[B26] Hönigsperger C, Marosi M, Murphy R, Storm JF (2015) Dorsoventral differences in Kv7/M-current and its impact on resonance, temporal summation and excitability in rat hippocampal pyramidal cells. J Physiol 593:1551–1580. 10.1113/jphysiol.2014.280826 25656084 PMC4386960

[B27] Hu H, Vervaeke K, Storm JF (2002) Two forms of electrical resonance at theta frequencies, generated by M‐current, h‐current and persistent Na+ current in rat hippocampal pyramidal cells. J Physiol 545:783–805. 12482886 10.1113/jphysiol.2002.029249 PMC2290731

[B28] Huang Z, Walker MC, Shah MM (2009) Loss of dendritic HCN1 subunits enhances cortical excitability and epileptogenesis. J Neurosci 29:10979–10988. 10.1523/JNEUROSCI.1531-09.2009 19726656 PMC2744118

[B29] Isokawa M (2000) Remodeling dendritic spines of dentate granule cells in temporal lobe epilepsy patients and the rat pilocarpine model. Epilepsia 41 [Suppl 6]:S14–S17. 10.1111/j.1528-1157.2000.tb01550.x 10999513

[B30] Jung S, Jones TD, Lugo JN, Sheerin AH, Miller JW, D'Ambrosio R, Anderson AE, Poolos NP (2007) Progressive dendritic HCN channelopathy during epileptogenesis in the rat pilocarpine model of epilepsy. J Neurosci 27:13012–13021. 18032674 10.1523/JNEUROSCI.3605-07.2007 PMC3087381

[B31] Jung S, Bullis JB, Lau IH, Jones TD, Warner LN, Poolos NP (2010) Downregulation of dendritic HCN channel gating in epilepsy is mediated by altered phosphorylation signaling. J Neurosci 30:6678–6688. 10.1523/JNEUROSCI.1290-10.2010 20463230 PMC2881658

[B32] Jung S, Warner LN, Pitsch J, Becker AJ, Poolos NP (2011) Rapid loss of dendritic HCN channel expression in hippocampal pyramidal neurons following status epilepticus. J Neurosci 31:14291–14295. 10.1523/JNEUROSCI.1148-11.2011 21976514 PMC3208968

[B33] Karschin C, Dissmann E, Stühmer W, Karschin A (1996) IRK(1-3) and GIRK(1-4) inwardly rectifying K channel mRNAs are differentially expressed in the adult rat brain. J Neurosci 16:3559–3570. 8642402 10.1523/JNEUROSCI.16-11-03559.1996 PMC6578832

[B34] Ketelaars SOM, Gorter JA, van Vliet EA, Lopes da Silva FH, Wadman WJ (2001) Sodium currents in isolated rat CA1 pyramidal and dentate granule neurones in the post-status epilepticus model of epilepsy. Neuroscience 105:109–120. 10.1016/S0306-4522(01)00176-2 11483305

[B35] Kim CS, Johnston D (2015) A1 adenosine receptor-mediated GIRK channels contribute to the resting conductance of CA1 neurons in the dorsal hippocampus. J Neurophysiol 113:2511–2523. 10.1152/jn.00951.2014 25652929 PMC4416607

[B36] Kjelstrup KG, Tuvnes FA, Steffenach H-A, Murison R, Moser EI, Moser M-B (2002) Reduced fear expression after lesions of the ventral hippocampus. Proc Natl Acad Sci USA 99:10825–10830. 10.1073/pnas.152112399 12149439 PMC125057

[B37] Liao YJ, Jan YN, Jan LY (1996) Heteromultimerization of G-protein-gated inwardly rectifying K+ channel proteins GIRK1 and GIRK2 and their altered expression in weaver brain. J Neurosci 16:7137–7150. 10.1523/JNEUROSCI.16-22-07137.1996 8929423 PMC6578936

[B38] Liu X, Muller RU, Huang L-T, Kubie JL, Rotenberg A, Rivard B, Cilio MR, Holmes GL (2003) Seizure-induced changes in place cell physiology: relationship to spatial memory. J Neurosci 23:11505–11515. 14684854 10.1523/JNEUROSCI.23-37-11505.2003 PMC6740937

[B39] Magee J (1999) Dendritic Ih normalizes temporal summation in hippocampal CA1 neurons. Nat Neurosci 2:508–514. 10.1038/12229 10461231

[B40] Malik R, Dougherty KA, Parikh K, Byrne C, Johnston D (2016) Mapping the electrophysiological and morphological properties of CA1 pyramidal neurons along the longitudinal hippocampal axis. Hippocampus 26:341–361. 10.1002/hipo.22526 26333017 PMC4760884

[B41] Marcelin B, Chauvière L, Becker A, Migliore M, Esclapez M, Bernard C (2009) h channel-dependent deficit of theta oscillation resonance and phase shift in temporal lobe epilepsy. Neurobiol Dis 33:436–447. 10.1016/j.nbd.2008.11.019 19135151

[B42] Marcelin B, Liu Z, Chen Y, Lewis AS, Becker A, McClelland S, Chetkovich DM, Migliore M, Baram TZ, Esclapez M, Bernard C (2012a) Dorsoventral differences in intrinsic properties in developing CA1 pyramidal cells. J Neurosci 32:3736–3747. 10.1523/JNEUROSCI.5870-11.2012 22423094 PMC3321843

[B43] Marcelin B, Lugo JN, Brewster AL, Liu Z, Lewis AS, McClelland S, Chetkovich DM, Baram TZ, Anderson AE, Becker A, Esclapez M, Bernard C (2012b) Differential dorso-ventral distributions of Kv4.2 and HCN proteins confer distinct integrative properties to hippocampal CA1 pyramidal cell distal dendrites. J Biol Chem 287:17656–17661. 10.1074/jbc.C112.367110 22511771 PMC3366832

[B44] McClelland S, Flynn C, Dubé C, Richichi C, Zha Q, Ghestem A, Esclapez M, Bernard C, Baram TZ (2011) Neuron-restrictive silencer factor-mediated hyperpolarization-activated cyclic nucleotide gated channelopathy in experimental temporal lobe epilepsy. Ann Neurol 70:454–465. 10.1002/ana.22479 21905079 PMC3177145

[B45] Milior G, Di Castro MA, Pepe’ Sciarria L, Garofalo S, Branchi I, Ragozzino D, Limatola C, Maggi L (2016) Electrophysiological properties of CA1 pyramidal neurons along the longitudinal axis of the mouse hippocampus. Sci Rep 6:1–9. 27922053 10.1038/srep38242 PMC5138623

[B46] Moser MB, Moser EI, Forrest E, Andersen P, Morris RG (1995) Spatial learning with a minislab in the dorsal hippocampus. Proc Natl Acad Sci USA 92:9697–9701. 7568200 10.1073/pnas.92.21.9697 PMC40869

[B47] Narayanan R, Johnston D (2007) Long-term potentiation in rat hippocampal neurons is accompanied by spatially widespread changes in intrinsic oscillatory dynamics and excitability. Neuron 56:1061–1075. 10.1016/j.neuron.2007.10.033 18093527 PMC2430016

[B48] Noam Y, Bernard C, Baram TZ (2011) Towards an integrated view of HCN channel role in epilepsy. Curr Opin Neurobiol 21:873–879. 10.1016/j.conb.2011.06.013 21782415 PMC3235400

[B49] Nolan MF, Malleret G, Dudman JT, Buhl DL, Santoro B, Gibbs E, Vronskaya S, Buzsáki G, Siegelbaum SA, Kandel ER, Morozov A (2004) A behavioral role for dendritic integration: HCN1 channels constrain spatial memory and plasticity at inputs to distal dendrites of CA1 pyramidal neurons. Cell 119:719–732. 10.1016/j.cell.2004.11.020 15550252

[B50] Oliveira MS, Skinner F, Arshadmansab MF, Garcia I, Mello CF, Knaus H-G, Ermolinsky BS, Otalora LFP, Garrido-Sanabria ER (2010) Altered expression and function of small-conductance (SK) Ca2+-activated K+ channels in pilocarpine-treated epileptic rats. Brain Res 1348:187–199. 10.1016/j.brainres.2010.05.095 20553876 PMC2916930

[B51] Pan Z, Kao T, Horvath Z, Lemos J, Sul JY, Cranstoun SD, Bennett V, Scherer SS, Cooper EC (2006) A common ankyrin-G-based mechanism retains KCNQ and NaV channels at electrically active domains of the axon. J Neurosci 26:2599–2613. 10.1523/JNEUROSCI.4314-05.2006 16525039 PMC6675151

[B52] Poolos NP, Migliore M, Johnston D (2002) Pharmacological upregulation of h-channels reduces the excitability of pyramidal neuron dendrites. Nat Neurosci 5:767–774. 10.1038/nn891 12118259

[B53] Powell KL, Ng C, O'Brien TJ, Xu SH, Williams DA, Foote SJ, Reid CA (2008) Decreases in HCN mRNA expression in the hippocampus after kindling and status epilepticus in adult rats. Epilepsia 49:1686–1695. 10.1111/j.1528-1167.2008.01593.x 18397293

[B54] Pyapali GK, Turner DA (1994) Denervation-induced dendritic alterations in CA1 pyramidal cells following kainic acid hippocampal lesions in rats. Brain Res 652:279–290. 7953741 10.1016/0006-8993(94)90238-0

[B55] Racine RJ (1972) Modification of seizure activity by electrical stimulation. II. Motor seizure. Electroencephalogr Clin Neurophysiol 32:281–294. 4110397 10.1016/0013-4694(72)90177-0

[B56] Racine RJ, Rose PA, Burnham WM (1977) Afterdischarge thresholds and kindling rates in dorsal and ventral hippocampus and dentate gyrus. Can J Neurol Sci 4:273–278. 10.1017/S0317167100025117 597802

[B57] Santoro B, Lee JY, Englot DJ, Gildersleeve S, Piskorowski RA, Siegelbaum SA, Winawer MR, Blumenfeld H (2010) Increased seizure severity and seizure-related death in mice lacking HCN1 channels. Epilepsia 51:1624–1627. 10.1111/j.1528-1167.2010.02554.x 20384728 PMC2952649

[B58] Schindelin J, Arganda-Carreras I, Frise E, Kaynig V, Longair M, Pietzsch T, Preibisch S, Rueden C, Saalfeld S, Schmid B, Tinevez J-Y, White DJ, Hartenstein V, Eliceiri K, Tomancak P, Cardona A (2012) Fiji: an open-source platform for biological-image analysis. Nat Methods 9:676–682. 10.1038/nmeth.2019 22743772 PMC3855844

[B59] Schramm J (2008) Temporal lobe epilepsy surgery and the quest for optimal extent of resection: a review. Epilepsia 49:1296–1307. 10.1111/j.1528-1167.2008.01604.x 18410360

[B76] Shah MM, Anderson AE, Leung V, Lin X, Johnston D (2004) Seizure-induced plasticity of h channels in entorhinal cortical layer III pyramidal neurons. Neuron 44:495–508. 10.1016/j.neuron.2004.10.011 15504329 PMC2386958

[B77] Shah MM, Huang Z, Martinello K (2013) HCN and KV7 (M-) channels as targets for epilepsy treatment. Neuropharmacology 69:75–81. 10.1016/j.neuropharm.2012.03.005 22446478 PMC4104618

[B60] Shin M, Brager D, Jaramillo TC, Johnston D, Chetkovich DM (2008) Mislocalization of h channel subunits underlies h channelopathy in temporal lobe epilepsy. Neurobiol Dis 32:26–36. 10.1016/j.nbd.2008.06.013 18657617 PMC2626192

[B61] Sholl DA (1953) Dendritic organization in the neurons of the visual and motor cortices of the cat. J Anat 87:387–406. 13117757 PMC1244622

[B62] Signorini S, Liao YJ, Duncan SA, Jan LY, Stoffel M (1997) Normal cerebellar development but susceptibility to seizures in mice lacking G protein-coupled, inwardly rectifying K^+^ channel GIRK2. Proc Natl Acad Sci USA 94:923–927. 10.1073/pnas.94.3.923 9023358 PMC19615

[B63] Singh NA, Charlier C, Stauffer D, DuPont BR, Leach RJ, Melis R, Ronen GM, Bjerre I, Quattlebaum T, Murphy JV, McHarg ML, Gagnon D, Rosales TO, Peiffer A, Anderson VE, Leppert M (1998) A novel potassium channel gene, KCNQ2, is mutated in an inherited epilepsy of newborns. Nat Genet 18:25–29. 10.1038/ng0198-25 9425895

[B64] Strange BA, Witter MP, Lein ES, Moser EI (2014) Functional organization of the hippocampal longitudinal axis. Nature Rev Neurosci 15:655–669. 10.1038/nrn3785 25234264

[B65] Su H, Sochivko D, Becker A, Chen J, Jiang Y, Yaari Y, Beck H (2002) Upregulation of a T-type Ca2+ channel causes a long-lasting modification of neuronal firing mode after status epilepticus. J Neurosci 22:3645–3655. 11978840 10.1523/JNEUROSCI.22-09-03645.2002 PMC6758371

[B66] Surges R, Freiman TM, Feuerstein TJ (2003) Gabapentin increases the hyperpolarization-activated cation current Ih in rat CA1 pyramidal cells. Epilepsia 44:150–156. 12558567 10.1046/j.1528-1157.2003.36802.x

[B67] Thom M, Liagkouras I, Martinian L, Liu J, Catarino CB, Sisodiya SM (2012) Variability of sclerosis along the longitudinal hippocampal axis in epilepsy: a post mortem study. Epilepsy Res 102:45–59. 10.1016/j.eplepsyres.2012.04.015 22608064 PMC3500681

[B68] Toyoda I, Bower MR, Leyva F, Buckmaster PS (2013) Early activation of ventral hippocampus and subiculum during spontaneous seizures in a rat model of temporal lobe epilepsy. J Neurosci 33:11100–11115. 10.1523/JNEUROSCI.0472-13.2013 23825415 PMC3718374

[B69] Vaidya SP, Johnston D (2013) Temporal synchrony and gamma-to-theta power conversion in the dendrites of CA1 pyramidal neurons. Nat Neurosci 16:1812–1820. 10.1038/nn.3562 24185428 PMC3958963

[B70] Wiebe S, Blume WT, Girvin JP, Eliasziw M, Effectiveness and Efficiency of Surgery for Temporal Lobe Epilepsy Study Group (2001) A randomized, controlled trial of surgery for temporal-lobe epilepsy. N Engl J Med 345:311–318. 10.1056/NEJM200108023450501 11484687

[B78] Wierschke S, Lehmann T-N, Dehnicke C, Horn P, Nitsch R, Deisz RA (2010) Hyperpolarization-activated cation currents in human epileptogenic neocortex. Epilepsia 51:404–414. 10.1111/j.1528-1167.2009.02275.x 19694789

[B71] Williams AD, Jung S, Poolos NP (2015) Protein kinase C bidirectionally modulates Ih and hyperpolarization-activated cyclic nucleotide-gated (HCN) channel surface expression in hippocampal pyramidal neurons. J Physiol 593:2779–2792. 10.1113/JP270453 25820761 PMC4506181

[B72] Wolfart J, Laker D (2015) Homeostasis or channelopathy? Acquired cell type-specific ion channel changes in temporal lobe epilepsy and their antiepileptic potential. Front Physiol 6:1178–23. 10.3389/fphys.2015.00168 PMC4467176 26124723

[B73] Wozny C, Gabriel S, Jandova K, Schulze K, Heinemann U, Behr J (2005) Entorhinal cortex entrains epileptiform activity in CA1 in pilocarpine-treated rats. Neurobiol Dis 19:451–460. 10.1016/j.nbd.2005.01.016 16023587

[B74] Yus-Nájera E, Muñoz A, Salvador N, Jensen BS, Rasmussen HB, Defelipe J, Villarroel A (2003) Localization of KCNQ5 in the normal and epileptic human temporal neocortex and hippocampal formation. Neuroscience 120:353–364. 12890507 10.1016/s0306-4522(03)00321-x

[B79] Zha Q, Brewster AL, Richichi C, Bender RA, Baram TZ (2008) Activity-dependent heteromerization of the hyperpolarization-activated, cyclic-nucleotide gated (HCN) channels: role of N-linked glycosylation. J Neurochem, 105:68–77. 10.1111/j.1471-4159.2007.05110.x 17988239 PMC2747799

